# Sarcopenic Obesity in Children: An Emerging Complication Evidenced by Clinical Data and a Juvenile Mouse Model

**DOI:** 10.1002/jcsm.70278

**Published:** 2026-04-17

**Authors:** Senjie Wang, Wei Zhang, Zhewen Qin, Xuelian Zhou, Chuqing Xue, Dan Wang, Xinyi Liang, Zixin Zhang, Shumin Zhan, Shan Wang, Wei Wu, Junfen Fu, Rahim Ullah

**Affiliations:** ^1^ Department of Endocrinology Children's Hospital Zhejiang University School of Medicine, National Clinical Research Center for Children and Adolescents' Health and diseases Hangzhou Zhejiang China

**Keywords:** childhood obesity, high‐fat diet, juvenile mouse model, muscle development, sarcopenic obesity, vitamin C

## Abstract

**Background:**

Sarcopenic obesity (SO) is well‐characterized in older adults, but its impact on muscle development in children remains poorly understood. This study investigated the effects of childhood obesity on musculoskeletal health and the underlying mechanisms.

**Methods:**

We enrolled 1447 children (31.4% girls; median age 11.10 years, IQR 9.39–12.50) for body composition assessment via dual‐energy X‐ray absorptiometry (DXA) and a separate cohort of 349 children (33.2% girls; median age 11.13 years, IQR 9.73–12.80) for grip strength measurement. A juvenile mouse model of high‐fat diet (HFD)‐induced obesity was established and compared to an adult‐onset model. Molecular pathways were examined via RNA sequencing and RT‐qPCR. Interventions included dietary reversal and vitamin C supplementation.

**Results:**

In children, the appendicular skeletal muscle mass ratio (ASMR) Z‐score was inversely correlated with BMI‐Z score (*ρ* = −0.369, *p* < 0.001) and body fat percentage (*ρ* = −0.668, *p* < 0.001). According to weight‐specific reference criteria, most of children with obesity exhibited low grip strength (below the 25th percentile). In mice, 4 weeks of HFD feeding in juveniles, but not adults, significantly reduced muscle mass (−6%, *p* < 0.05), muscle fibre diameter (−8%, *p* < 0.001), grip strength (−14%, *p* < 0.01) and rotarod performance (−29%, *p* < 0.05). RNA sequence and RT‐qPCR revealed that HFD suppressed myogenic regulatory factors (e.g., *Myod*, *Myog*) and promoted adipogenic pathways specifically in juveniles. In stark contrast, adult mice showed no such impairments after 4 weeks of HFD. The muscle deficits caused by juvenile obesity were not resolved but persisted even after subsequent dietary weight loss. Vitamin C supplementation effectively mitigated HFD‐induced impairments, increasing muscle fibre diameter (~15%, *p* < 0.001, vs. HFD), grip strength (~10%, *p* < 0.001, vs. HFD) and expression of key myogenic genes (e.g., *Myod*, *Myog*, all *p* < 0.05).

**Conclusions:**

Childhood obesity critically impairs muscle development during the juvenile growth period, driven by transcriptomic reprogramming that suppresses myogenesis. These deficits are persistent and not reversed by weight loss alone. Vitamin C supplementation presents a potential therapeutic strategy to protect muscle health in children with obesity. The juvenile mouse model established herein provides a novel tool for future research into childhood SO.

**Abbreviations**
Adipoq: adiponectin
ASM: appendicular skeletal muscle mass
ASMI: appendicular skeletal muscle mass index
ASMR: appendicular skeletal muscle mass ratio
BIA: bioelectrical impedance analysis
BMI: body mass index
Btg2: B‐cell translocation gene 2
DEGs: differentially expressed genes
DMD: Duchenne muscular dystrophy
DXA: Dual‐energy X‐ray absorptiometry
ECM: extracellular matrix
Fabp4: fatty acid binding protein 4
Fos: Fos proto‐oncogene
GEO: Gene Expression Omnibus
GO: Gene Ontology
H&E: haematoxylin and eosin
HFD: high‐fat diet
IMLA: intramuscular lipid accumulation
IQR: interquartile range
MHCs: myosin heavy chains
MRFs: myogenic regulatory factors
MRI: magnetic resonance imaging
Myf5: myogenic factor 5
Myf6: myogenic factor 6
Myh1: myosin heavy chain 1
Myh2: myosin heavy chain 2
Myh4: myosin heavy chain 4
Myh7: myosin heavy chain 7
Myod: myogenic differentiation 1
Myog: myogenin
NCD: normal chow diet
Nr4a1: nuclear receptor subfamily 4 group A member 1
Pax7: paired box 7
Ppar‐γ: peroxisome proliferator‐activated receptor gamma
RNA‐Seq: RNA sequencing
RT‐qPCR: real‐time quantitative polymerase chain reaction
SC: satellite cell
SEM: standard error of the mean
Skil: SKI‐like proto‐oncogene
SO: sarcopenic obesity
TA: tibialis anterior

## 1 Introduction

Patients with obesity often exhibit reduced muscle mass and function, a health issue that has received increasing attention in older populations. It is estimated that approximately 11% of older adults with obesity worldwide are affected by sarcopenic obesity (SO) [1]. However, standardized diagnostic and therapeutic guidelines for paediatric SO are currently lacking, despite growing recognition of the condition [2, 3]. Reported prevalence rates of SO in children and adolescents range from 5.7% to 69.7% in girls and from 7.2% to 81.3% in boys [4]. This wide variation highlights the absence of uniform assessment methods for muscle mass and function in this population, highlighting the urgent need for more basic and clinical research to elucidate the underlying mechanisms and identify effective clinical management strategies for childhood SO.

Muscle mass and muscle function are two crucial indicators of muscular health [5]. Reliable methods for assessing muscle mass include dual‐energy X‐ray absorptiometry (DXA), bioelectrical impedance analysis (BIA) and magnetic resonance imaging (MRI) [6, 7]; various methods exist for evaluating muscle function, such as handgrip strength, standing long jump and sit‐ups; among these, handgrip strength is a practical and easily implementable clinical test [8, 9]. The appendicular skeletal muscle mass index (ASMI) is a key metric for assessing muscle mass in the diagnostic criteria for SO in adults [10]. Unlike the well‐defined diagnostic cut‐offs for adults (e.g., DXA‐derived ASMI < 7.0 kg/m^2^ for men and < 5.4 kg/m^2^ for women), establishing diagnostic criteria for SO in children and adolescents is challenging due to their dynamic growth and development and the scarcity of large‐scale reference data on muscle mass in healthy children. Several studies have proposed population‐specific reference values based on age and sex [11, 12, 13], involving metrics such as the appendicular skeletal muscle mass ratio (ASMR), ASMI and muscle mass index (MMI). While the clinical accuracy and applicability of these indices in paediatric practice remain to be consolidated [14], they represent significant progress in the field. Similarly, defining appropriate diagnostic cut‐offs for handgrip strength is difficult. A recent study reported weight‐specific grip strength reference curves derived from 4072 healthy Chinese children and adolescents aged 6–18 years [15], providing a valuable reference standard for grip strength assessment in this population. Collectively, these studies lay a foundational basis for assessing muscle mass and function in children with obesity.

Existing mouse or rat models of SO typically require prolonged high‐fat diet (HFD) feeding (often greater than 6 months) to induce the phenotype of reduced muscle mass [16, 17]. This approach is based on the concept of a ‘double hit’ combining aging and nutritional stress [18], where the internal factor of aging and the external factor of HFD synergize to rapidly and prominently induce a classic SO phenotype. However, juvenile rodents lack the intrinsic factor of aging, and there is a conflict between their short juvenile period and the relatively long HFD exposure typically required to induce significant muscle impairment. To the best of our knowledge, a validated juvenile animal model of SO has not yet been established.

This study begins with clinical investigation, enrolling children with normal BMI and obesity to analyse the relationship between childhood obesity and muscle mass/function using DXA for body composition and Jamar dynamometers for upper limb grip strength. We then established a juvenile mouse model of obesity and compared it with an adult‐onset obesity model to dissect the specific impact of HFD‐induced obesity on muscle development during the juvenile period. Finally, we investigated whether weight loss through dietary intervention and a targeted nutritional intervention (e.g., Vitamin C supplementation) could ameliorate muscle developmental impairments in juvenile obese mice.

## 2 Materials and Methods

### 2.1 Study Participants

This study was approved by the Ethics Committee of the Children's Hospital of Zhejiang University School of Medicine (Approval number: 2024‐IRB‐0208‐P‐01) and conducted in accordance with the principles of the Declaration of Helsinki. Written informed consent was obtained from all participants and their parents or legal guardians. The clinical component of this study comprises two independent cohorts:

1. Muscle mass assessment cohort: A retrospective study including children and adolescents who underwent body composition analysis via dual‐energy X‐ray absorptiometry (DXA) at our hospital between 2016 and 2024.
2. Muscle strength assessment cohort: A cross‐sectional study recruiting participants from the endocrinology outpatient clinic between April and October 2024, during which body composition and grip strength were measured concurrently.

### 2.2 Anthropometric Measurements

Body weight was measured to the nearest 0.1 kg using calibrated electronic scales, with participants in light clothing without shoes. Height was measured to the nearest 0.1 cm using a stadiometer, with participants standing upright without shoes. Body mass index (BMI) was calculated as weight in kilograms divided by height in meters squared. Height, weight and BMI values were converted to age‐ and sex‐specific standard deviation scores (SDS) based on World Health Organization reference data [S1]. Waist circumference was measured to the nearest 0.1 cm at the midpoint between the lowest rib and the iliac crest using a non‐stretch tape. Pubertal development was assessed by paediatric endocrinologists using the Tanner staging system [S2].

### 2.3 Muscle Mass Assessment

Body composition, including fat mass, lean mass and appendicular skeletal muscle mass (ASM), was assessed using a DXA scanner (Hologic Discovery Wi, USA) following the standard procedures of the International Society for Clinical Densitometry [S3]. This analysis included all children and adolescents who underwent DXA at our hospital from 2016 to 2024. Participants were excluded based on pre‐defined criteria: (i) presence of skeletal dysplasia; (ii) missing essential anthropometric or DXA data; (iii) medical conditions known to severely affect muscle mass (e.g., spinal muscular atrophy, severe malnutrition); or (iv) declined participation in the hospital's research program. Consequently, 1447 participants were included in the final muscle mass analysis.

To assess relative muscle mass, the ASMR was calculated as follows:

$$\begin{array}{c} \text{Appendicular skeletal muscle mass ratio}\;\left(\text{ASMR}\right)=\\ \;\frac{\text{Appendicular muscle mass}\;\left(\mathrm{kg}\right)}{\text{Weight}\;\left(\mathrm{kg}\right)}\times 100\% \end{array}$$

To account for the effects of age and sex, individual ASMR values were converted to Z‐scores (ASMR‐Z) using reference values established for Chinese children and adolescents by Liu et al. [13].

### 2.4 Muscle Strength Assessment

Upper limb grip strength was assessed using a Jamar plus^+^ dynamometer (ASP Global LLC). With the participant in a standing position and the elbow fully extended, two trials were performed with the dominant hand, and the maximum value (kg) was recorded. To enable standardized comparison, grip strength was evaluated using weight‐specific reference curves, which were derived from a large cohort of 4072 healthy Chinese children and adolescents [15]. This reference defines low grip strength as performance below the 25th percentile (P25). Each participant's measured grip strength was compared against the sex‐ and weight‐matched P25 reference value to determine adequacy.

Body composition for this cohort was additionally assessed via bioelectrical impedance analysis (BIA) using an InBody S10 analyser (Biospace Co. Ltd., South Korea). Metrics obtained included body fat mass, fat‐free mass and muscle mass.

For this arm of the study, 439 participants were initially recruited from the endocrinology outpatient clinic between April and October 2024. Application of the exclusion criteria—(i) physical disability or surgically implanted devices; (ii) age < 6 or > 18 years; (iii) conditions affecting muscle function (e.g., spinal muscular atrophy, dermatomyositis, malnutrition); (iv) recent acute illness; or (v) missing data—resulted in a final sample of 349 children for muscle strength analysis. Childhood obesity was defined according to Chinese national standards [S4]. The final cohort consisted of 93 normal‐weight children and 256 children with obesity.

### 2.5 Animal Care and Grouping

All experimental procedures complied with the National Institutes of Health Guidelines for the care and use of laboratory animals and received approval from the Animal Care and Use Committee of Zhejiang University (ethics code: ZJU20210313). C57BL/6JGpt mice (Strain No. N000013), aged 3 and 8 weeks, were procured from GEMPHARMATECH in Shanghai, China. These mice were accommodated at the Zhejiang University animal facility, maintained under a 12‐h light/dark cycle and provided ad libitum access to food and water. The mice were fed either a normal chow diet (NCD, D12450B, Research Diets Inc., New Brunswick, New Jersey, USA) consisting of 67.3% carbohydrates (70% calories), 19.2% protein (20% calories) and 4.3% fat (10% calories) or an HFD (12492i, Research Diets Inc.) comprising 26.3% carbohydrates (20% calories), 26.2% protein (20% calories) and 34.9% fat (60% calories). The 3‐week‐old and 8‐week‐old C57BL/6J wild‐type mice were distributed into four groups based on diet and age: Juvenile‐NCD group, Juvenile‐HFD group, Adult‐NCD group and Adult‐HFD group, with each group containing eight mice (Figure 2A). For dietary intervention, juvenile‐HFD mice were switched from an HFD to an NCD at 8 weeks of age and maintained on the NCD for an additional 5 weeks, until they reached 13 weeks of age. In contrast, juvenile‐NCD mice continued on the NCD until they also reached 13 weeks. These groups were designated as the HC and CC groups, respectively (Figure 5A). For vitamin C intervention, 24 randomly selected mice were equally divided into three groups: NCD + Water, HFD + Water and HFD + Vitamin C, with the latter receiving vitamin C (6 g/L, A4544‐25G, Sigma‐Aldrich) in their drinking water. The drinking water was refreshed every other day until the conclusion of the experiment (Figure 7A). Only male mice were used in all experiments, and they were randomly assigned to each experimental group.

### 2.6 Assessment of Food Consumption and Protein Intake

Food intake experiments were performed with both NCD and HFD. Mice were housed individually and allowed to acclimatize for 24 h before data collection. An electronic balance with a precision of 0.001 g was used to weigh food, while body weight was determined using a separate balance accurate to 0.01 g. Each animal was provided with a pre‐weighed quantity of diet; after 24 h, the uneaten portion was re‐weighed, and consumption was derived by subtracting the residual from the initial weight. Measurements were carried out over 48 h, and the mean daily intake (g/day) was determined. The energy density was 1.41 kcal/g for NCD and 5.21 kcal/g for HFD. The protein content by weight was 19.2% in NCD and 26.2% in HFD. Daily energy intake (kcal) was computed as: Energy intake = Food intake (g) × Energy density of the diet (kcal/g). Daily protein intake (g) was calculated as: Protein intake = Food intake (g) × Protein content of the diet (weight %).

### 2.7 Assessment of Body Composition in Mice

For the mice, body composition assessment, encompassing both fat and lean mass, was performed using a low‐field nuclear magnetic resonance (NMR) instrument (model QMR06‐090H, manufactured by Suzhou Niumag Analytical Instrument Corporation, China).

### 2.8 Haematoxylin–Eosin (H&E) Staining in Mice

Tibialis anterior (TA) muscle tissue was fixed in 4% paraformaldehyde, and paraffin‐embedded sections (3 μm thick) were prepared. The sections were then stained with H&E following standard protocols [S5]. The slides were then sealed with neutral gum. The stained tissue sections were examined under a microscope (Eclipse Ci‐L, Nikon, Japan), with images captured and analysed using Image‐Pro Plus 6.0 software (Media Cybernetics, USA).

### 2.9 Oil Red O Staining in Mice

Oil Red O staining was used to visualize lipid accumulation. The process began with staining frozen sections of fixed TA muscle tissues with Oil Red O for 8–10 min while shielding them from light. The sections were then washed using standard procedures [S6]. The quality of the staining was assessed under a microscope to ensure proper visualization of lipids. Successful staining was followed by image acquisition and analysis using Image‐Pro Plus 6.0 software.

### 2.10 Assessment of Grip Strength in Mice

Grip strength test in mice was performed using a grip strength meter (Model SA417, Sansbio, China). To conduct the test, mice were allowed to grasp the induction crossbar of the grip strength meter. Once the animals secured a grip on the crossbar, they were gently pulled backward horizontally by the tail until their grip loosened. The peak force exerted just before their grip was released was recorded as the absolute grip strength of the mice's limbs.

### 2.11 Panlab Rotarod Test in Mice

The rotarod test was conducted to evaluate the motor performance of mice using a rotating horizontal rod (Sansbio, Jiangsu, China) in a controlled light and quiet environment. Prior to the main experiment, mice underwent a pre‐training session the day before, which involved two 5‐min trials at a speed of 4 rpm on the rotarod apparatus. On the day of the test, the mice were initially set on the rod at a steady speed of 4 rpm for 10 s. Subsequently, the speed was incrementally increased from 4 rpm to 40 rpm over a period of 300 s. Each mouse performed the test three times, and the average duration they remained on the rod across these three trials was recorded as the result.

### 2.12 Open Field Test in Mice

The open field test was conducted using an open Plexiglas box measuring 45 cm × 45 cm × 45 cm, with an illuminance of 250 lx at the centre [S7]. Mice were placed in one corner of the apparatus and permitted to explore freely for a duration of 5 min. During this time, the total distance travelled and the average speed of each mouse were recorded to assess their locomotor activity and exploratory behaviour. To maintain consistent conditions and prevent olfactory cues from influencing the behaviour of subsequent test subjects, the apparatus was thoroughly cleaned with 75% ethanol between each test.

### 2.13 RNA Sequencing and Data Analysis in Mice

TA muscle tissues were harvested from mice and immediately snap‐frozen in liquid nitrogen. Total RNA was isolated from the muscle using FreeZol reagent (R711‐01, Vazyme), following the manufacturer's protocol, and eluted in 20 μL of sterile water. Bulk RNA sequencing was performed by Novogene Co. Ltd. (Beijing, China). Briefly, mRNA was purified from total RNA using poly‐T oligo‐attached magnetic beads. Fragmentation was achieved using divalent cations at elevated temperatures in First Strand Synthesis Reaction Buffer (5×). First‐strand cDNA was synthesized using random hexamer primers and M‐MuLV Reverse Transcriptase, followed by RNA degradation with RNase H. Second‐strand cDNA synthesis was performed using DNA Polymerase I and dNTP. The cDNA was processed to create blunt ends through exonuclease/polymerase activities, followed by 3′ adenylation and ligation of adaptors with a hairpin loop structure. Library fragments were purified using the AMPure XP system (Beckman Coulter, Beverly, USA) and amplified by PCR. The library was quantified using both a Qubit 2.0 Fluorometer and an Agilent 2100 Bioanalyzer. After confirming the insert size, qRT‐PCR was performed to accurately determine the effective concentration of the library, ensuring a concentration greater than 2‐nM Sequencing was conducted on the Illumina NovaSeq 6000 platform, generating 150 bp paired‐end reads. Sequencing utilized the Sequencing by Synthesis (SBS) method with four fluorescently labelled dNTPs, DNA polymerase and sequencing primers. Raw sequencing data were processed on the Galaxy platform (https://usegalaxy.org/). Quality control was performed using FastQC (version 0.12.1), and low‐quality reads were trimmed using Cutadapt (version 4.9). After quality control, reads were aligned to the mouse reference genome (GRCm38/mm10) using RNA STAR (version 2.7.11a) [S8]. Gene expression was quantified with featureCounts (version 2.0.3) [S9], referencing Gencode version 20 annotations [S10]. Differential expression analysis was carried out using the DESeq2 package (version 1.42.0) in R (version 4.2.2), with genes identified as differentially expressed genes (DEGs) based on a |log2FC| > 0.5 and *p*‐value < 0.05. Pathway enrichment analysis, including Gene Ontology (GO) terms, was performed using the ClusterProfiler package (version 4.10.1) in R. Venn diagrams for data visualization were generated using an online tool [S11].

### 2.14 RNA Isolation and Real‐Time Quantitative PCR (RT‐qPCR) in Mice

Total RNA was extracted from the TA muscle of mice using FreeZol reagent (R711‐01, Vazyme), following the manufacturer's protocol. The isolated RNA was then reverse‐transcribed to cDNA utilizing the HiScript II Q RT SuperMix (R222‐01, Vazyme). RT‐qPCR was conducted using ChamQ Universal SYBR qPCR Master Mix (Q711‐02, Vazyme). The relative expression levels of the target genes were quantified using the 2^−△△Ct^ method, with GAPDH serving as the internal control to normalize the expression of target genes, as validated in models [S12, S13]. The specific RT‐qPCR primers used were detailed in Table S1.

### 2.15 Statistical Analyses

For statistical analyses, various software tools were utilized, including GraphPad Prism (version 8.01, GraphPad Software), SPSS (version 24, IBM SPSS Statistics) and R (version 4.5.1).

For the clinical data, descriptive data are presented as medians with interquartile ranges (IQR) for continuous variables due to their non‐normal distributions, whereas categorical variables are expressed as frequencies and percentages. Differences between the two groups (Non‐SO vs. SO) were assessed using the Mann–Whitney *U* test for continuous variables and Fisher's exact test for categorical variables. In animal experiments, outliers were first excluded using the Iterative Grubbs test. Normality of the data was evaluated using the Shapiro–Wilk test, while homogeneity of variances was assessed with Levene's test or Mauchly's test of sphericity. Data comparisons were performed using an unpaired two‐tailed Student's *t*‐test for normally distributed data and either an unpaired two‐tailed Mann–Whitney rank‐sum test or a Wilcoxon signed‐rank test for non‐normally distributed data. For comparisons among three or more groups with normally distributed data and equal variances, one‐way or two‐way ANOVA was used, followed by Sidak's post hoc test. For comparisons involving three or more groups with non‐normally distributed data, the Kruskal–Wallis one‐way ANOVA on ranks was applied, followed by a Bonferroni post hoc test. The investigators conducted experiments and analyses while blinded to the conditions to ensure unbiased results. All data were presented as means ± SEM. A *p*‐value of less than 0.05 was deemed statistically significant.

## 3 Results

### 3.1 Appendicular Skeletal Muscle Mass Ratio Is Inversely Associated With the Degree of Obesity in Children

This study retrospectively enrolled children and adolescents aged 3–18 years who underwent dual‐energy X‐ray absorptiometry (DXA) scanning at our hospital's endocrinology department between 2016 and 2024. After applying pre‐defined exclusion criteria (e.g., missing data, skeletal dysplasia), a total of 1447 participants were included in the muscle mass analysis (participant characteristics are shown in Table S2). The median age of this cohort was 11.10 years (IQR: 9.39–12.50), with boys comprising 68.6%. As DXA scans were primarily indicated for obesity assessment, the overall prevalence of obesity in this study population was high. The median BMI was 27.97 kg/m^2^ (IQR: 25.54–31.02), and the median BMI‐Z score was 2.93 (IQR: 2.51–3.49). Regarding body composition, the median fat mass was 6.431 kg (IQR: 5.218–8.230), median body fat percentage was 45.50% (IQR: 41.74%–49.56%) and the median ASMR was 22.68% (IQR: 20.81%–24.62%).

**FIGURE 1:**
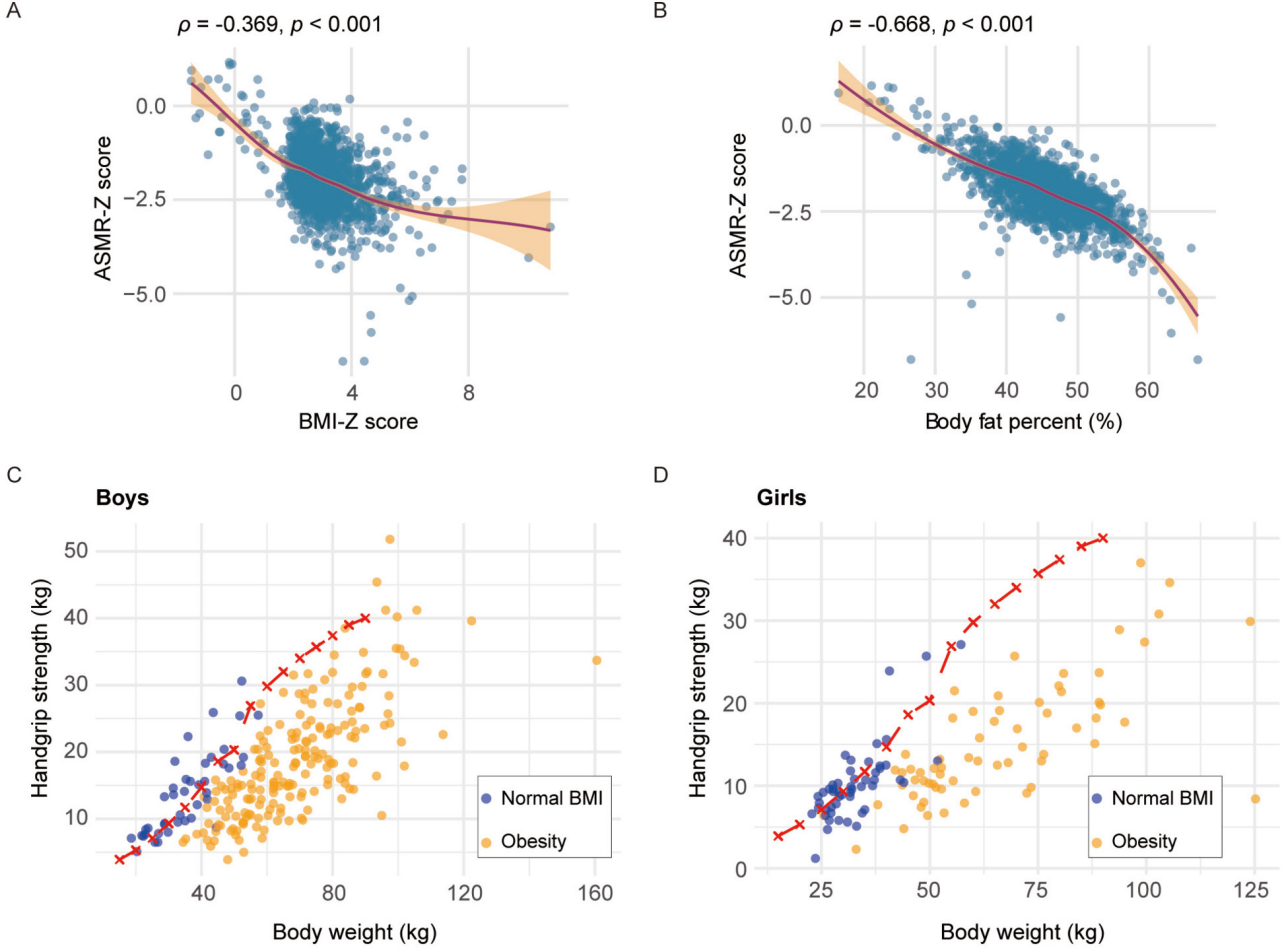
Assessment of muscle mass and grip strength in children and adolescents. (A and B) Spearman correlation analysis between the Z‐score of appendicular skeletal muscle mass ratio (ASMR) and BMI‐Z score (A) and body fat percentage (B) in 1447 children and adolescents (A: *ρ* = −0.369, *p* < 0.001; B: *ρ* = −0.668, *p* < 0.001). The ASMR‐Z score was calculated based on published reference values from 10 818 healthy Chinese children, matched for sex and age [13]. (C and D) Grip strength distribution in 349 children and adolescents, stratified by boys (C) and girls (D). The red dotted lines represent the 25th percentile (P25) of weight‐specific grip strength derived from a reference cohort of 4072 healthy Chinese children [15]. Data points below this line indicate low grip strength for a given body weight.

We conducted Spearman's rank correlation analysis to evaluate ASM in relation to BMI‐Z score and body fat percentage. Absolute ASM showed a non‐monotonic association with BMI‐Z score (*ρ* = −0.019, *p* = 0.474; Figure S1A) and body fat percentage (*ρ* = −0.266, *p* < 0.001; Figure S1B), with higher absolute muscle mass observed in most children with obesity compared to normal‐weight children. The recently updated Asian Working Group for Sarcopenia guidelines propose the ASM/BMI index as a novel diagnostic criterion [14]. Applying this index to our paediatric cohort, we observed a negative correlation with BMI‐Z score (*ρ* = −0.363, *p* < 0.001; Figure S1C) and an even stronger inverse association with body fat percentage (*ρ* = −0.467, *p* < 0.001; Figure S1D). The ASMR is recommended for assessing muscle quality in children [7]. To account for the effects of age and sex, individual ASMR values were converted to Z‐scores (ASMR‐Z score) using reference values for Chinese children and adolescents established by Liu et al. [13]. Spearman's rank correlation analysis was used to assess the associations of ASMR‐Z score with BMI‐Z score and body fat percentage. The results revealed a negative correlation between ASMR‐Z score and BMI‐Z score (*ρ* = −0.369, *p* < 0.001, Figure 1A) and a stronger negative correlation with body fat percentage (*ρ* = −0.668, *p* < 0.001, Figure 1B).

### 3.2 Upper Limb Muscle Strength Is Generally Inadequate in Children With Obesity

To further investigate whether this reduced muscle mass ratio translates into functional impairment, we next assessed grip strength. The cross‐sectional study was conducted prospectively from April to October 2024, recruiting children and adolescents aged 6–18 years from our hospital's endocrinology outpatient clinic and measuring their upper limb grip strength and body composition. Based on inclusion and exclusion criteria, data from 349 participants were analysed (Table S3). The median age of this cohort was 11.13 years (IQR: 9.73–12.80), with boys comprising 66.8%. According to paediatric obesity diagnostic criteria, 256 children were classified into the obesity group and 93 into the normal BMI group. Given the significant differences in age and sex distribution between two groups (both *p* < 0.001, Table S3), a fair comparison was made using the sex‐ and weight‐specific grip strength reference curves for healthy Chinese children proposed by Li et al. [15]. This study recommends using weight‐specific percentile curves, with the 25th percentile (P25) as the cut‐off point for low grip strength. Grip strength results showed that the vast majority of children with obesity (both boys and girls) had grip strength values below the P25 reference line corresponding to their sex and weight (Figure 1C,D). In contrast, children in the normal BMI group generally exhibited higher grip strength levels than the obesity group, with most values lying above the P25 reference line (Figure 1C,D). This indicates that children and adolescents with obesity commonly exhibit inadequate upper limb grip strength relative to their body weight.

### 3.3 Four Weeks of High‐Fat Diet Feeding Induces Obesity and Impairs Lean Mass and Muscle Function in Juvenile Mice

**FIGURE 2:**
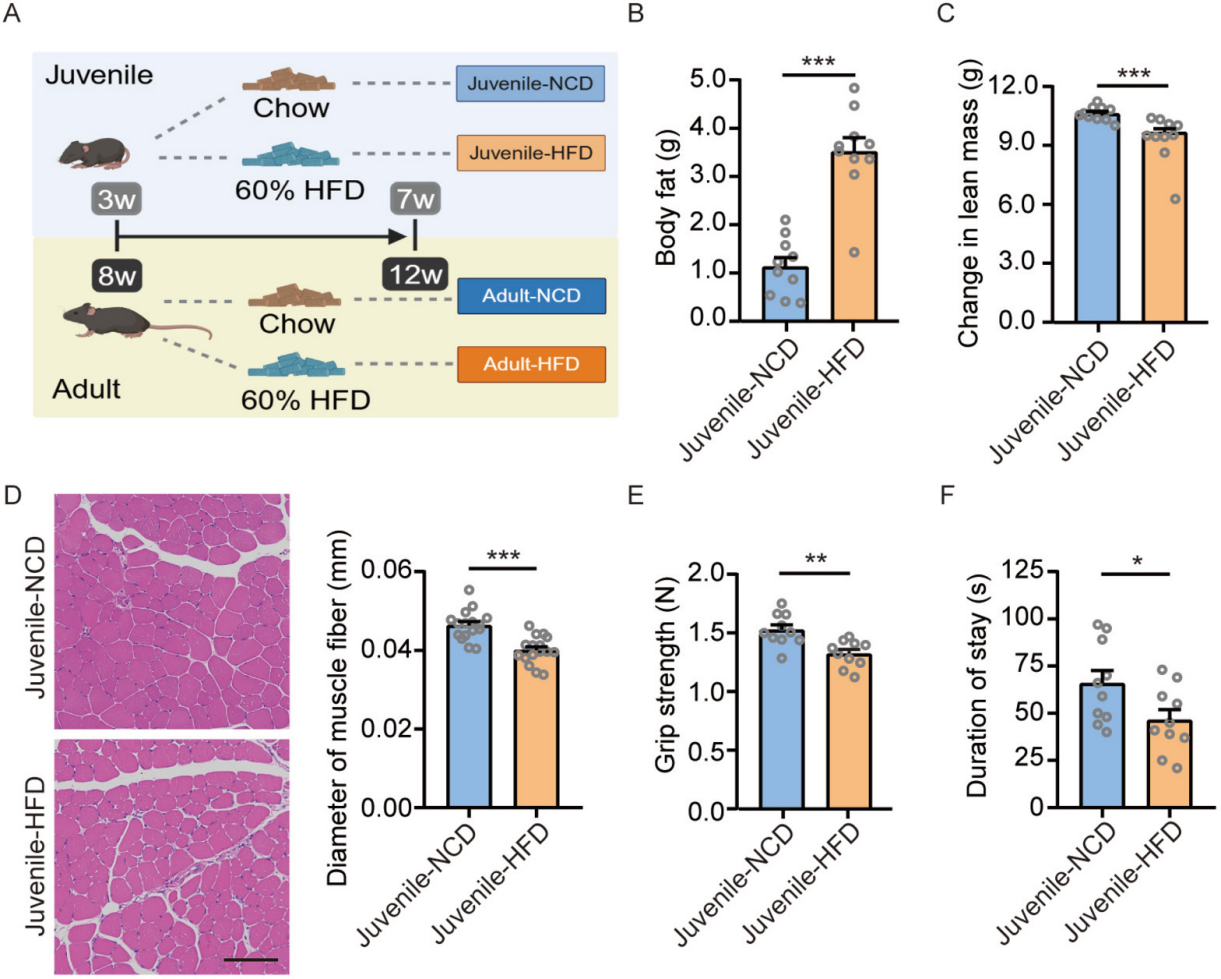
Four weeks of high‐fat diet feeding induces an obese phenotype and concomitant impairments in muscle mass and function in 3‐week‐old weanling mice. (A) Schematic diagram of the feeding regimens for the juvenile and adult obesity mouse models. (B) Body fat mass of juvenile mice at 7 weeks of age (*n* = 10 mice/group). (C) Change in lean mass in juvenile mice from 3 to 7 weeks of age (*n* = 10 mice/group). (D) Representative H&E‐stained sections of TA muscles from juvenile mice (scale bar = 100 μm), with quantification of muscle fibre diameter (*n* = 8 mice/group). (E) Muscle strength assessed by grip strength test in juvenile mice (*n* = 10 mice/group). (F) Motor performance assessed by the rotarod test in juvenile mice with latency to fall (*n* = 10 mice/group). Values are presented as mean ± SEM *p < 0.05, **p < 0.01, ***p < 0.001.

To investigate whether childhood obesity during the developmental stage causally impairs muscle, we established a juvenile mouse model by exposing 3‐week‐old post‐weaning mice to a 60% HFD for 4 weeks (Figure 2A). Food, energy and protein intake were assessed at 3 and 7 weeks of age. Compared with controls, HFD‐fed mice exhibited lower 24‐h food intake (Figure S2A) but higher total energy intake after caloric normalization (Figure S2B). Protein intake, calculated based on dietary protein content, did not differ between groups (Figure S2C). Body composition was assessed at 7 weeks of age. Analysis revealed significant increases in body weight (Figure S2D), total body fat mass (Figure 2B) and body fat percentage (Figure S2E) in HFD‐fed mice compared to NCD controls. Fat‐free mass was not significantly different between groups (Figure S2F), but both total lean mass (Figure S2G) and the gain in lean mass from 3 to 7 weeks of age (Figure 2C) were significantly lower in the HFD group.

To further explore the structural and functional consequences of HFD‐induced obesity in juvenile mice, we performed histological and functional assessments of skeletal muscles. H&E staining revealed that HFD feeding reduced the diameter (Figure 2D) and average cross‐sectional area (Figure S2H) of muscle fibres in the TA muscle of juvenile mice. To evaluate muscle function, we conducted grip strength tests to measure muscle strength and the rotarod test to assess motor coordination and performance. HFD feeding resulted in reductions in absolute grip strength (Figure 2E), as well as relative grip strength normalized to either body weight or lean mass (Figure S2I,J). Furthermore, in the rotarod test, HFD‐fed mice exhibited impaired motor performance, indicated by significant reductions in both the latency to fall (Figure 2F) and the maximum speed achieved on the rotating rod (Figure S2K). We subsequently assessed general locomotor activity using the open field test, which revealed no significant differences between the HFD and NCD groups (Figure S2L,M).

### 3.4 Four Weeks of High‐Fat Diet Feeding Induces Obesity but Does Not Affect Lean Mass or Muscle Function in Adult Mice

Next, we aimed to determine whether similar muscular alterations occur in adult mice after 4 weeks of HFD feeding. Eight‐week‐old mice were fed an HFD for 4 weeks, and their body composition was analysed at 12 weeks of age (Figure 2A). While HFD feeding similarly resulted in increased body weight (Figure S3A), body fat mass (Figure S3B) and body fat percentage (Figure S3C) in adult mice, it did not significantly alter fat‐free mass (Figure S3D), total lean mass (Figure S3E) or the gain in lean mass during this period (Figure S3F). H&E staining showed that HFD feeding did not influence the diameter or average cross‐sectional area of muscle fibres in adult mice (Figure S3G–I). Paradoxically, in adult mice, HFD feeding increased both absolute grip strength (Figure S3J) and relative grip strength normalized to lean mass (Figure S3L). However, no significant effects were observed for grip strength normalized to body weight (Figure S3K), latency to fall or maximum speed on the rotarod (Figure S3M,N) or for total distance travelled and average speed in the open field test (Figure S3O,P). Collectively, these results indicate that 4 weeks of HFD feeding does not impair muscle mass or function in adult mice.

### 3.5 Four Weeks of High‐Fat Diet Feeding Impairs Muscle Development in Juvenile Mice

**FIGURE 3:**
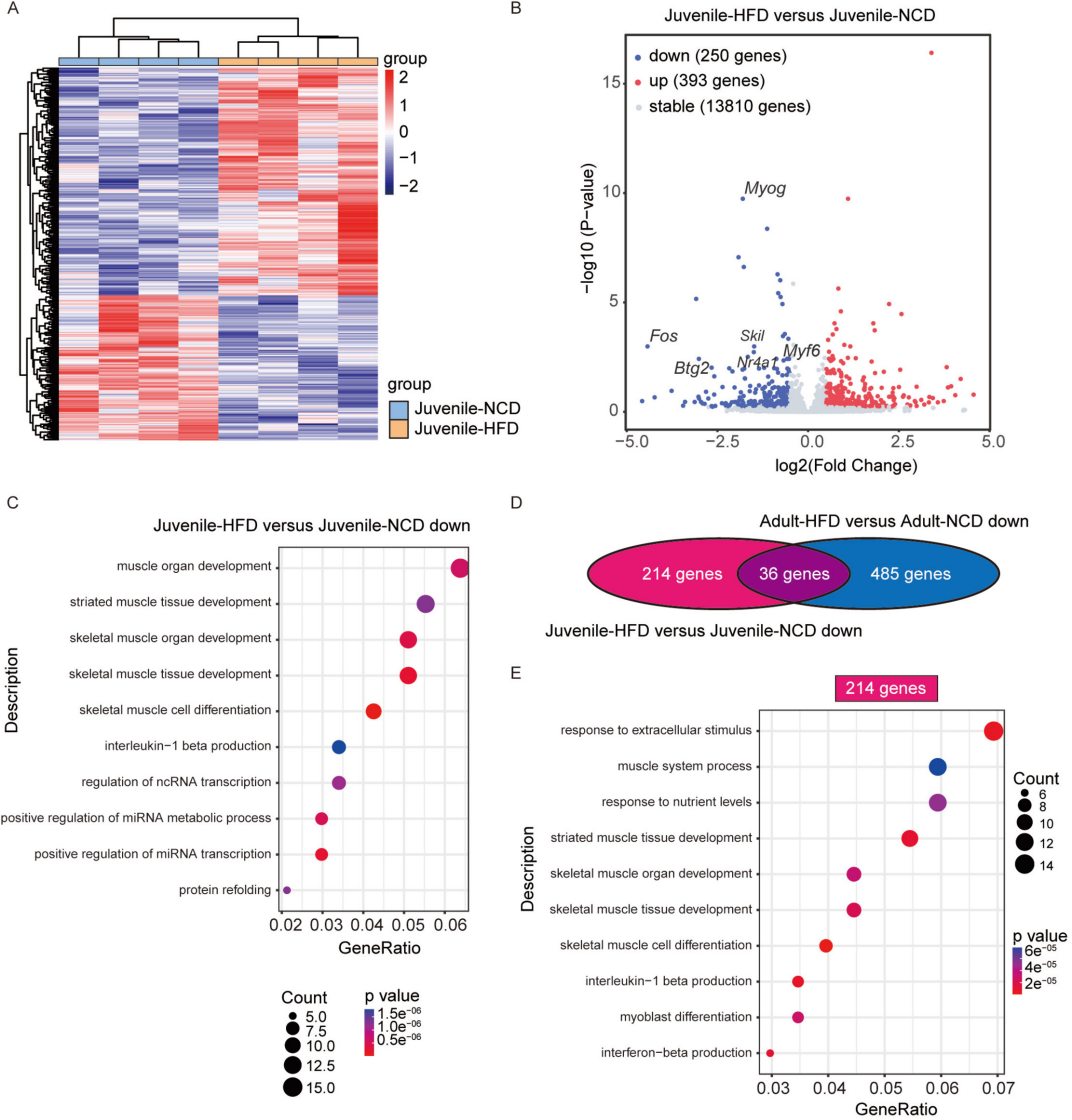
RNA‐Seq analysis of TA muscles from juvenile mice. (A) Heatmap showing differentially expressed genes (DEGs) in juvenile HFD‐fed mice compared to juvenile NCD‐fed mice. (B) Volcano plot displaying 393 upregulated (red) and 250 downregulated (blue) genes in the Juvenile‐HFD group versus the Juvenile‐NCD group. (C) GO enrichment analysis of biological processes for the downregulated genes in the Juvenile‐HFD group compared to the Juvenile‐NCD group. (D) Venn diagram depicting the overlap of downregulated DEGs between juvenile and adult HFD‐fed mice compared to their respective NCD controls. (E) GO enrichment analysis of biological processes for the 214 juvenile‐specific downregulated DEGs. Data are presented from *n* = 4 mice per group.

To further evaluate the effects of an HFD on muscle tissue at the molecular level, we performed RNA sequencing analysis on muscle tissues from HFD‐fed and NCD‐fed juvenile and adult mice. Genomic analysis identified a total of 643 differentially expressed genes (DEGs) in juvenile HFD‐fed mice compared to NCD‐fed controls, with 393 genes upregulated and 250 genes downregulated (Figure 3A,B). Notably, Gene Ontology (GO) enrichment analysis revealed that the upregulated genes were predominantly involved in lipid oxidation and metabolic pathways, whereas the downregulated genes were primarily associated with muscle development processes (Figure 3C). In adult mice, 854 DEGs were identified in response to HFD feeding, comprising 333 upregulated and 521 downregulated genes compared to the NCD group (Figure S4A,B). Similar to juveniles, upregulated genes in adults were enriched for lipid oxidation and metabolism. However, the downregulated genes in adults were uniquely associated with extracellular matrix (ECM) organization and cell‐substrate adhesion (Figure S4C).

We subsequently performed an overlap analysis to identify shared and unique DEGs between HFD‐fed juvenile and adult mice compared to their respective NCD controls (Figure 3D). Among the upregulated DEGs, both juvenile and adult HFD groups exhibited enhanced pathways related to lipid metabolism. Regarding the downregulated DEGs, 214 juvenile‐specific downregulated DEGs were predominantly linked to muscle development processes (Figure 3E). In contrast, 485 adult‐specific downregulated DEGs were mainly involved in extracellular structure and matrix organization, which were associated with connective tissue processes (Figure S4D).

To elucidate age‐specific gene expression patterns under basal conditions, we compared muscle transcriptomes between NCD‐fed adult and juvenile mice. This analysis identified 1646 DEGs, with 560 genes upregulated and 1086 genes downregulated in adults relative to juveniles (Figure S5A,B). GO enrichment analysis indicated that the upregulated genes in adult mice were associated with amino acid metabolism and xenobiotic metabolic processes (Figure S5C). Conversely, the downregulated genes were enriched for biological processes related to cell proliferation, differentiation and growth‐associated signalling pathways (Figure S5D). This differential expression profile underscores distinct age‐specific molecular adaptations. Collectively, these transcriptomic profiles indicate that HFD feeding in juvenile mice, but not in adults, disrupts the genetic programs essential for muscle development.

**FIGURE 4:**
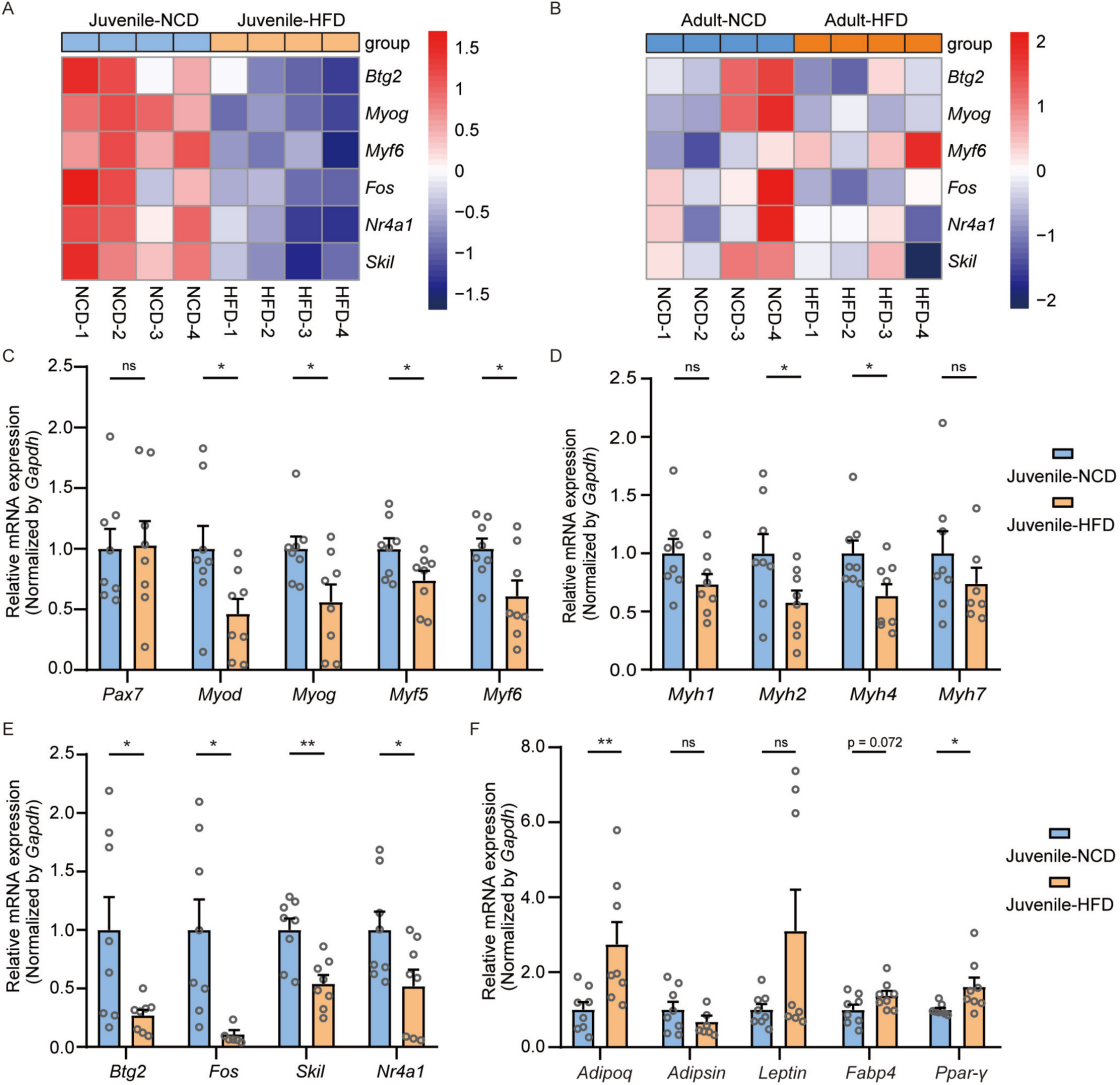
Juvenile HFD‐fed mice exhibit suppressed myogenic and enhanced adipogenic gene expression. (A and B) Heatmap showing expression of myogenic genes in juvenile and adult mice (*n* = 4 mice/group). (C–F) Gene expression levels in the TA muscle of juvenile mice (*n* = 8 mice/group unless noted otherwise): (C) mRNA levels of myogenic regulatory factors (MRFs); (D) mRNA levels of myosin heavy chains (MHCs; for *Myh7*, Juvenile‐HFD: *n* = 7); (E) mRNA levels of potential myogenic regulators (for *Fos*, Juvenile‐HFD: *n* = 6); (F) mRNA levels of adipogenic factors (for *Adipsin*, Juvenile‐HFD: *n* = 7). Values are mean ± SEM *p < 0.05, **p < 0.01, ***p < 0.001.

RNA‐Seq analysis revealed that HFD feeding downregulated genes associated with muscle development, with distinct expression patterns between juvenile and adult mice (Figure 4A,B). To validate the impairment of muscle development in juvenile obese mice, we performed RT‐qPCR to examine key regulatory genes. Results confirmed significant downregulation of crucial myogenic regulatory factors (MRFs), including *Myf5*, *Myod*, *Myog* and *Myf6*, in juvenile HFD‐fed mice compared to NCD controls (Figure 4C), indicating compromised muscle growth. We next examined the expression of myosin heavy chains (MHCs), key structural components of muscle fibres. Juvenile HFD‐fed mice showed markedly reduced expression of *Myh2* and *Myh4* (markers of type II muscle fibres), while *Myh1* and *Myh7* expression remained unaltered (Figure 4D). Furthermore, expression of potential upstream regulators of MRFs and MHCs—including *Btg2*, *Fos*, *Skil* and *Nr4a1*—was also decreased in HFD‐fed juvenile mice (Figure 4A,E). Given the association between intramuscular lipid accumulation (IMLA) and impaired muscle structure and function [19], we assessed the expression of key adipokines and adipogenic factors. HFD‐fed juvenile mice exhibited elevated mRNA levels of *Adipoq* (*adiponectin*), *Leptin*, *Fabp4* and *Ppar‐γ*, whereas *Adipsin* expression was unchanged (Figure 4F). Consistent with this pro‐adipogenic shift, Oil Red O staining of muscle sections revealed a significantly larger lipid‐positive area in the HFD group compared to NCD controls (Figure S6A,B).

In contrast, HFD feeding in adult mice did not significantly affect the expression of MRFs (Figure S7A), MHCs (Figure S7B), *Btg2*, *Fos*, *Skil*, *Nr4a1* (Figure S7C) or most adipogenic factors except for *Adipoq* (Figure S7D). Collectively, these findings demonstrate that an HFD impairs muscle growth and function in juvenile mice by downregulating key myogenic factors and promoting intramuscular adipogenesis, whereas these effects are largely absent in adult mice.

### 3.6 HFD‐Induced Muscle Impairments in Juvenile Mice Persist After Weight Loss Through Dietary Intervention

**FIGURE 5:**
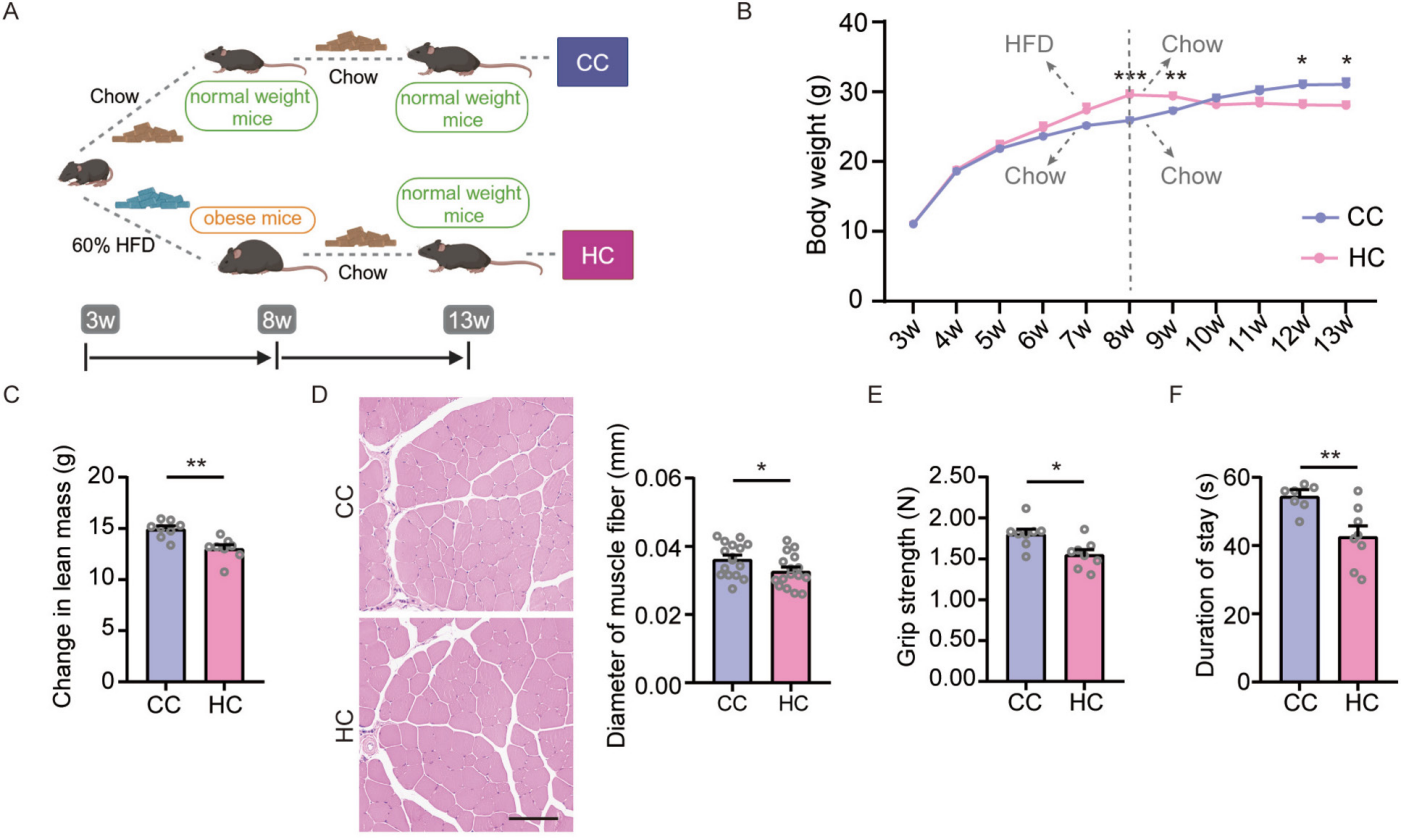
Dietary intervention failed to reverse the HFD‐induced deficits in muscle mass and function. (A) Schematic of the dietary intervention protocol. (B) Body weight change from 3 to 13 weeks of age. (C) Change in lean mass in juvenile mice from 3 to 13 weeks of age. (D) Representative H&E‐stained sections of TA muscles (scale bar = 100 μm), with quantification of muscle fibre diameter. (E) Muscle strength assessed by grip strength test. (F) Motor performance assessed by the rotarod test with latency to fall. *n* = 8 mice/group. Values are mean ± SEM *p < 0.05, **p < 0.01, ***p < 0.001.

**FIGURE 6:**
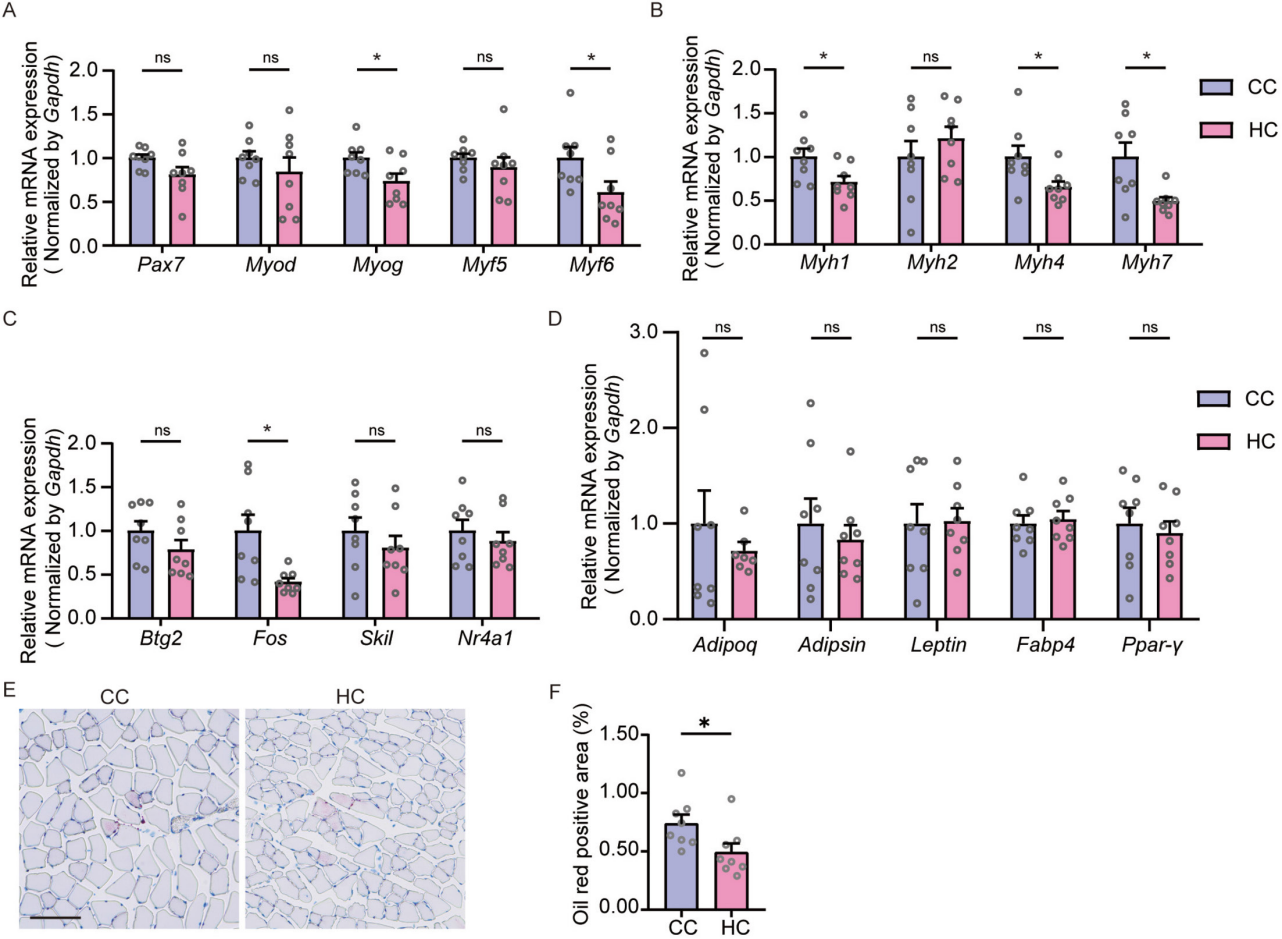
Dietary intervention failed to correct HFD‐induced abnormalities in molecular signalling pathways in muscle. (A–D) mRNA expression levels in TA muscle (*n* = 8/group; for Adipoq, HC: *n* = 7): myogenic regulatory factors (A), myosin heavy chains (B), potential myogenic regulators (C) and adipogenic factors (D). (E and F) Representative Oil Red O staining of TA muscle (scale bars = 100 μm, *n* = 8/group) and quantification of lipid area. Values are mean ± SEM *p < 0.05, **p < 0.01, ***p < 0.001.

Given that the HFD‐induced muscular impairments during juvenility appear to be persistent, we sought to determine whether a dietary intervention initiated after the establishment of obesity could reverse these deficits. Juvenile mice initially fed an HFD were switched to an NCD at 8 weeks of age (HC group) and maintained on this diet until 13 weeks (Figure 5A). At 13 weeks, the HC group exhibited significantly lower body weight than mice continuously fed an NCD (CC group) (Figure 5B), confirming successful weight loss. Body composition analysis showed that total body fat and body fat percentage were comparable between the CC and HC groups (Figure S8A,B). However, the HC group displayed significant reductions in fat‐free mass, absolute lean mass and lean mass gain compared to the CC group (Figures S8C,D and 5C). Histological and functional assessments revealed persistent muscular deficits. H&E staining showed reduced muscle fibre diameter and cross‐sectional area in the HC group (Figures 5D and S8E). While grip strength normalized to body or lean weight showed no significant difference (Figure S8F,G), absolute grip strength was lower in the HC group (Figure 5E). Furthermore, the HC group performed worse on the rotarod test, with decreased latency to fall and maximum speed (Figures 5F and S8H), indicating sustained impairments in muscle strength and motor coordination. General locomotor activity in the open field test was unaffected (Figure S8I,J). Molecular analysis revealed downregulation of key myogenic genes in the HC group. The *Myog* was significantly reduced, along with *Myf6* (Figure 6A). Expression of myosin heavy chains *Myh1*, *Myh4* and *Myh7* was also significantly lower (Figure 6B). Potential upstream regulators, including *Fos*, showed decreased expression, though changes in *Btg2*, *Skil* and *Nr4a1* were not statistically significant (Figure 6C). In contrast, adipogenic factors and intramuscular lipid accumulation (assessed by Oil Red O staining) were normalized in the HC group (Figure 6D–F).

These findings indicate that while dietary intervention successfully reversed body weight, fat mass and intramuscular adipogenesis, it failed to restore the structural, functional and key molecular deficits in muscle induced by juvenile obesity.

### 3.7 Vitamin C Supplementation Mitigates HFD‐Induced Muscular Impairments in Juvenile Mice

**FIGURE 7:**
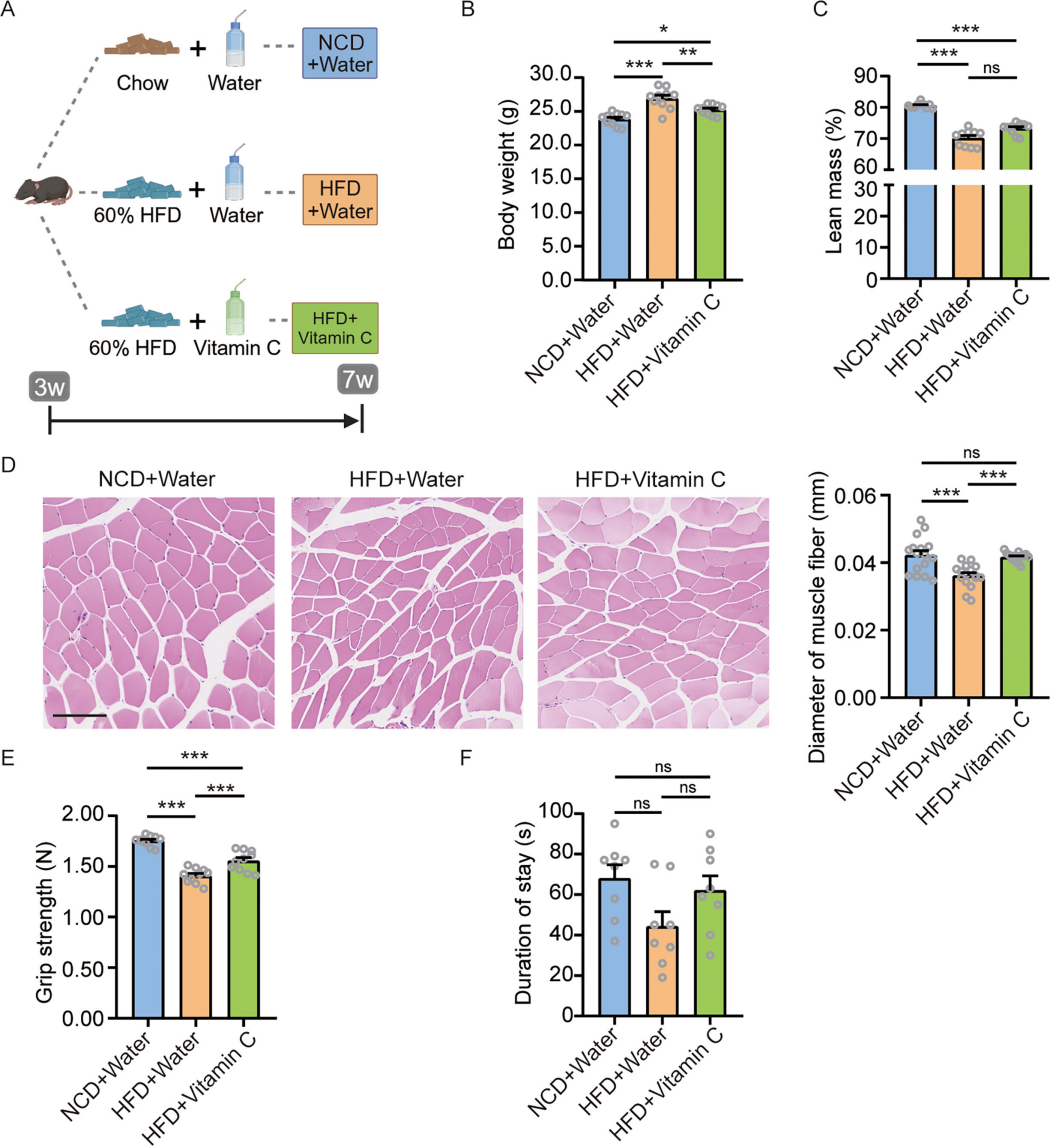
Vitamin C supplementation ameliorated the HFD‐induced impairment of muscular function. (A) Experimental timeline. (B and C) Body weight and lean mass percent at 13 weeks of age (*n* = 10/group). (D) Representative H&E‐stained sections of TA muscles (scale bar = 100 μm), with quantification of muscle fibre diameter (*n* = 8/group). (E) Muscle strength assessed by grip strength test (*n* = 8/group). (F) Motor performance assessed by the rotarod test with latency to fall (*n* = 8/group). Data are mean ± SEM *p < 0.05, **p < 0.01, ***p < 0.001.

To directly test whether the impaired muscle development in our model was mechanistically reversible, we employed vitamin C as a proof‐of‐concept intervention based on its established role in activating the MyoD/Myog pathway [20, 21, 22]. Juvenile mice were fed an HFD with or without vitamin C supplementation and compared to NCD controls (Figure 7A). Body composition, muscle histology, gene expression and functional parameters were assessed. Vitamin C significantly attenuated HFD‐induced body weight gain, though its effects on total fat mass, body fat percentage, fat‐free mass and lean mass did not reach statistical significance (Figures 7B,C and S9A–D). Histologically, vitamin C prevented the HFD‐induced reduction in muscle fibre diameter and cross‐sectional area (Figures 7D and S9E). Functionally, it improved absolute grip strength and grip strength normalized to both body weight and lean mass (Figures 7E and S9F,G). A non‐significant improvement in rotarod performance was also observed (Figures 7F and S9H), while general locomotor activity remained comparable across groups (Figure S9I,J).

**FIGURE 8:**
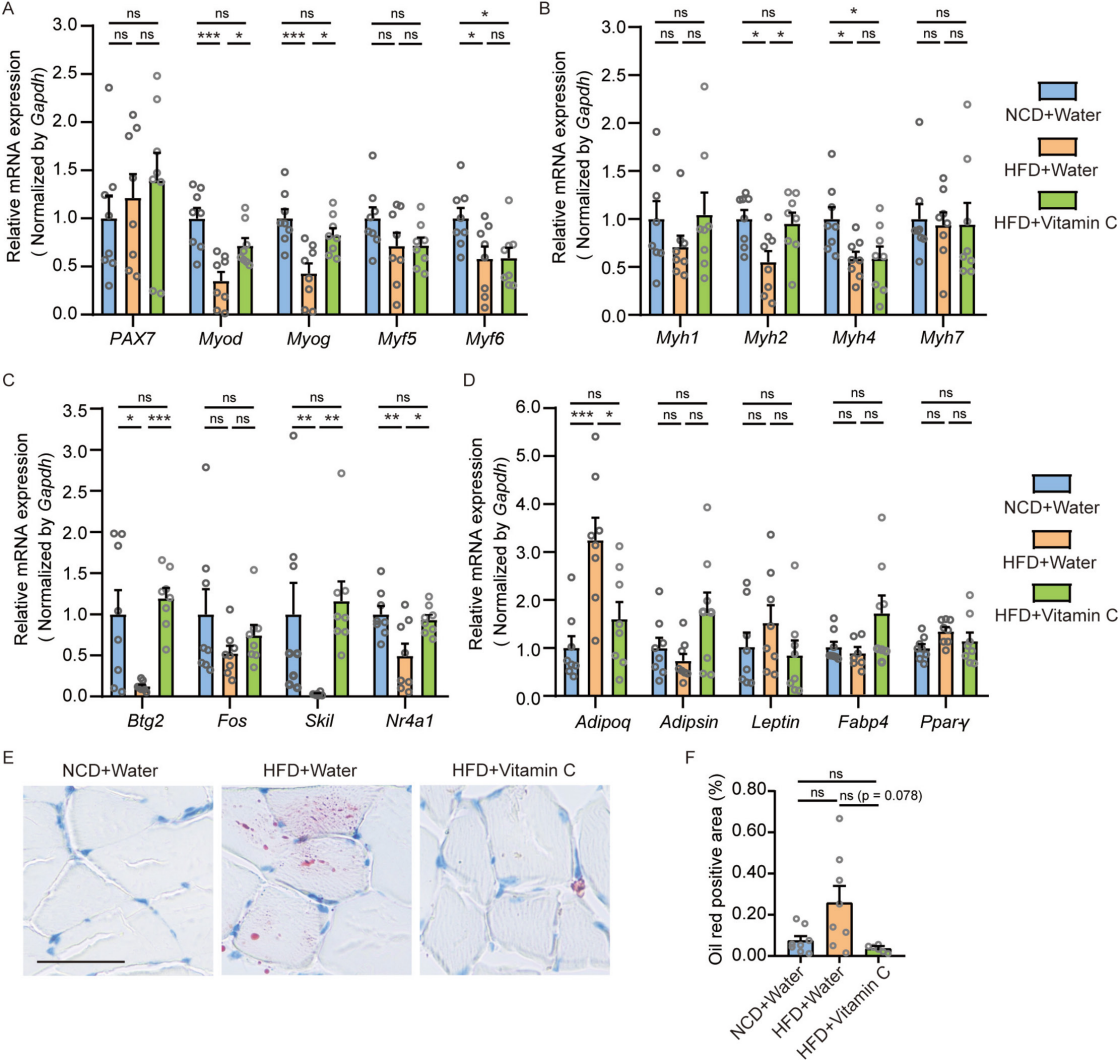
Vitamin C modulates myogenic and adipogenic gene expression. (A–D) mRNA levels in TA muscle (*n* = 8/group unless noted): (A) Myogenic regulatory factors (MRFs). (B) Myosin heavy chains (MHCs). (C) Potential myogenic regulators (for *Skil*, HFD + Water group: *n* = 7). (D) Adipogenic factors (for *FABP4*, HFD + Water group: *n* = 7). (E and F) Representative Oil Red O staining (scale bars = 100 μm; HFD + Vitamin C group: *n* = 6) and lipid area quantification. Data are mean ± SEM *p < 0.05, **p < 0.01, ***p < 0.001.

At the molecular level, vitamin C significantly increased the expression of the myogenic regulators *Myod* and *Myog* (Figure 8A) and upregulated the myosin heavy chain isoforms *Myh1* and *Myh2* (Figure 8B). It also elevated the expression of potential upstream regulators *Btg2*, *Skil* and *Nr4a1* (Figure 8C). Concurrently, vitamin C reduced intramuscular lipid accumulation, as evidenced by a decreased Oil Red O‐positive area (Figure 8E,F), and significantly downregulated *Adipoq* expression (Figure 8D).

In summary, vitamin C supplementation alleviated HFD‐induced muscular impairments by enhancing the expression of key myogenic factors and reducing intramuscular adipogenesis, highlighting its potential as a supportive intervention for muscle health in juvenile obesity.

## 4 Discussion

This study integrates clinical and animal model research to demonstrate the detrimental impact of childhood obesity on musculoskeletal health, characterized by significant reductions in relative muscle mass and function. Crucially, we identify the developmental period as a unique window of susceptibility to obesity‐induced myopathy. In a juvenile mouse model of HFD‐induced obesity, we observed impaired muscle growth and function, closely associated with transcriptomic reprogramming in muscle tissue featuring suppressed myogenic programs and activated adipogenic pathways. These muscular deficits, triggered by juvenile‐onset obesity, were persistent and failed to reverse even after successful weight loss through dietary intervention. However, vitamin C supplementation emerged as a viable therapeutic strategy, effectively mitigating these impairments. Our findings indicate that childhood obesity impairs muscle development by altering myogenic pathways and underscore the urgency of early intervention to protect muscle health in children with obesity.

In paediatric clinical practice, the diagnosis of sarcopenic obesity remains constrained by the absence of standardized muscle metrics and validated age‐specific cut‐offs [14]. Absolute muscle mass is unsuitable as a standalone metric due to variations across sex, age and pubertal stage, as reflected by its non‐monotonic distribution in our cohort. Our analysis shows that reduced ASMR in obesity is driven by increased adiposity, not muscle loss. Both ASM/BMI and ASMR Z‐score showed significant negative correlations with BMI‐Z score and body fat percentage. ASMR, which accounts for the dilution of muscle by adiposity, may be a more sensitive metric for paediatric sarcopenic obesity [7]. While ASM/BMI complements adult diagnostic criteria by better detecting muscle loss in severe obesity [10], its application in children requires further validation. The strength per unit of muscle is not fundamentally impaired in obesity, but higher body weight imposes greater mechanical load. A low muscle‐to‐weight ratio may increase the risk of functional limitation and injury, warranting multidimensional studies on muscle strength.

Clinical studies have established that obesity adversely affects muscle structure and function in both adults and children [4, 23]. Consistent with these observations, our study identified a similar pattern in children and juvenile mice with obesity: relative muscle mass, grip strength and motor performance were significantly compromised. Importantly, our findings extend beyond these traditional associations by revealing differences in relative muscle mass and muscle strength among individuals with the same BMI or body weight. This highlights that muscle‐related indicators, rather than BMI or body weight alone, play a pivotal role in assessing children's muscular health. The decline in relative muscle function is particularly concerning, as it is directly linked to reduced physical activity, potentially exacerbating obesity and its associated complications in a vicious cycle [2, 24]. Longitudinal evidence further indicates that muscular fitness in childhood predicts future cardiometabolic and bone health outcomes independently of BMI [25]. Thus, focusing solely on weight reduction without addressing muscle quality may overlook a central pathway through which obesity perpetuates metabolic and functional decline, especially across various weight‐loss interventions. Our results strongly suggest that assessing and improving relative muscle function should be accorded importance equal to weight reduction in the management of paediatric obesity. This is especially critical in neuromuscular disorders such as Duchenne muscular dystrophy (DMD), where long‐term corticosteroid use and reduced mobility predispose patients to obesity. Our data demonstrate suppression of myogenic regulators in juvenile obesity, which may exacerbate regenerative failure and accelerate functional decline. This aligns with clinical evidence linking obesity in DMD to an increased risk of fractures and obstructive sleep apnoea, thereby perpetuating immobilization and metabolic dysfunction [S14, S15]. Accordingly, obesity management in DMD should prioritize preservation of muscle health in addition to weight control.

A key finding of this study is the divergent response to obesity‐induced muscle injury between juvenile and adult periods. Aligning with reports that short‐term HFD feeding has minimal effect or may even slightly improve muscle metrics in adult mice [26, 27, 28], we found that adult mice exhibited no significant impairments in lean mass, muscle structure or function after 4 weeks of HFD. This disparity likely originates from fundamental differences in satellite cell (SC) dynamics across ages. In rodents, the SC pool diminishes and the cell cycle lengthens with age [29, 30]. SCs in juveniles are highly proliferative, supporting ongoing muscle growth, whereas in adults, they are predominantly quiescent, activated mainly in response to injury or exercise. Consequently, the numerical superiority and proliferative activity of SCs in juveniles may render them more vulnerable to the metabolic stress imposed by HFD, leading to more pronounced damage. Our RNA‐Seq data provide direct molecular evidence for this age‐specific susceptibility. In juvenile HFD‐fed mice, downregulated genes were significantly enriched in muscle development processes. In contrast, downregulated genes in adult HFD‐fed mice were primarily associated with extracellular matrix organization, potentially laying the groundwork for longer‐term muscle pathology. This suggests that HFD directly targets the core transcriptional program governing muscle development in juvenile mice. We confirmed the significant downregulation of key MRFs (*Myf5*, *MyoD*, *Myog*, *Myf6*) via RT‐qPCR. These factors form the core network governing the myogenic cascade: *Myf5* and *MyoD* determine myogenic commitment, *Myog* directs terminal differentiation and *Myf6* not only oversees myofiber maturation but also helps maintain the SC pool via epidermal growth factor release [31, 32, 33]. Their coordinated downregulation is likely a major contributor to the observed impairment of muscle growth.

Furthermore, we observed decreased expression of several potential upstream regulators (*Btg2*, *Fos*, *Skil*, *Nr4a1*). These factors play critical roles in positively regulating myogenesis: *Btg2* promotes the expression of MRFs and MHCs while inhibiting adipogenesis [34]; *Fos*, a component of the AP‐1 transcription complex, interacts with *MyoD* in a feedback loop influencing the balance between myocyte proliferation and differentiation [35, 36]; *Skil* counteracts TGF‐β signalling‐mediated suppression of *MyoD* activity and myocyte fusion [S16–S18]; and *Nr4a1*, an orphan nuclear receptor whose deficiency impairs embryonic and postnatal muscle development, reduces MRF and MHC expression and promotes adipocyte progenitor proliferation [37, 38]. The downregulation of these upstream regulators collectively constitutes an upstream molecular event in HFD‐mediated suppression of juvenile muscle development. Analysis of the MHC expression profile further revealed the fibre‐type specificity of HFD‐induced damage. Expression of *Myh1*, *Myh2* and *Myh4* (encoding MHC‐IIX, MHC‐IIA and MHC‐IIB, respectively, markers of the predominant fast‐twitch glycolytic fibres in TA muscle [S19]) was significantly reduced, while *Myh7* (encoding MHC‐I, a slow‐twitch oxidative fibre marker) remained unchanged. This pattern, consistent with findings in adult HFD‐fed mice [S20], suggests that HFD selectively damages fast‐twitch fibres, which may form the structural basis for the observed decreases in muscle strength and power.

The adverse health effects of childhood obesity can persist into adulthood [S21]. Our dietary intervention experiment uncovered a critical finding: Despite the normalization of body weight, body fat, IMLA and adipogenic factor expression, the structural, functional and molecular deficits in muscle induced by juvenile obesity persisted. This ‘metabolic memory’ effect suggests that metabolic stress during a critical developmental window may lead to persistent deficits to muscle developmental potential, possibly via epigenetic reprogramming. This finding carries major clinical significance: It emphasizes that for childhood obesity, intervention cannot be delayed. Measures to protect muscle health must be implemented early; otherwise, lifelong motor dysfunction and metabolic risk may ensue, especially considering muscle's role as the primary site for postprandial glucose disposal [39].

Based on the known beneficial effects of vitamin C on muscle health [20, 21, 22], we explored its interventional potential. Encouragingly, vitamin C effectively alleviated various HFD‐induced muscular impairments. Mechanistically, vitamin C not only directly upregulated key MRFs like *MyoD* and *Myog* and structural proteins like *Myh1* and *Myh2*, but, more importantly, it reversed the downregulation of the upstream regulators *Btg2*, *Skil* and *Nr4a1*. Given that these factors positively regulate MRFs and MHCs, vitamin C likely exerts its protective effects by activating this upstream regulatory network [34, 40]. Concurrently, vitamin C suppressed the aberrant increase in adipogenic factors like *Adipoq* and reduced IMLA, thereby improving the muscle metabolic microenvironment. These results highlight the potential of vitamin C as a candidate for translational research aimed at mitigating obesity‐associated myopathy in children.

This study has several limitations. First, our clinical cohort lacked detailed puberty stage and hormonal measurements, and our juvenile mouse model does not fully encompass the evolving endocrine landscape of human puberty (e.g., variations in GH, IGF‐1 and testosterone), which is known to modulate myogenic factors [S22, S23]. Second, the specific molecular mechanisms linking HFD feeding to the downregulation of the identified upstream regulators (Btg2, Fos, Skil, Nr4a1) require further detailed investigation. Third, the optimal dosage and timing for vitamin C intervention in children remain to be determined. Finally, the cross‐sectional design of our clinical component inherently limits causal inference. While we observed strong inverse associations between obesity indices and muscle mass and function, this design cannot disentangle whether the observed muscular impairments are a primary consequence of obesity‐related metabolic stress or a secondary result of reduced physical activity, which may both coexist in a reinforcing cycle. Future research could focus on: (1) undertaking prospective, longitudinal studies that simultaneously track muscle health and obesity progression in children and incorporate assessments of pubertal stage and hormone profiles to clarify causal mechanisms; (2) tracking the long‐term impact of childhood muscle impairment on metabolic health in adulthood; (3) elucidating the precise upstream signals (e.g., inflammation, lipotoxicity) through which HFD disrupts the myogenic/adipogenic balance; (4) validating the beneficial effects of vitamin C or other potentially effective drugs on muscle function in clinical cohorts of children with obesity.

Our findings establish the juvenile growth period as a critical window of vulnerability to obesity‐induced myopathy. The persistence of muscle deficits even after dietary weight loss underscores that early identification of impaired muscle development should prompt proactive and integrated intervention strategies aimed at preserving muscle quality. These approaches should include structured exercise regimens, such as resistance training to stimulate myogenesis; tailored nutritional support combining low‐fat diets with specific nutrients like vitamin C, which in our model mitigated the obesity‐driven suppression of *Myod* and *Myog*; and potential pharmacological agents designed to enhance myogenic signalling or reduce intramuscular adiposity. The juvenile mouse model presented here provides a directly translatable pre‐clinical platform for evaluating such combined strategies. The protective effect of vitamin C serves as a key proof‐of‐concept, demonstrating the model's utility for screening candidate interventions.

## Funding

This study was supported by the National Natural Science Foundation of China (Nos. 82350410491 and 82370863), Key R&D Program of Zhejiang (No. 2023C03047), the National Key R&D Program of China (No. 2021YFC2701900) and the Research Fund of National Health Commission and Zhejiang Major Health Science and Technology (WKJ‐ZJ‐2535).

## Ethics Statement

This study was conducted in accordance with the Declaration of Helsinki and approved by the Medical Ethics Committee of the Children's Hospital, Zhejiang University School of Medicine (Approval number: 2024‐IRB‐0208‐P‐01). Informed consent was obtained from all subjects involved in the study. All experimental procedures complied with the National Institutes of Health Guidelines for the care and use of laboratory animals and received approval from the Animal Care and Use Committee of Zhejiang University (ethics code: ZJU20210313).

## Conflicts of Interest

The authors declare no conflicts of interest.

## Supporting information


**Table S1:** RT‐qPCR primer list.
**Table S2:** Characteristic of the participants for muscle mass assessment.
**Table S3:** Characteristic of the participants for muscle function assessment.
**Figure S1:** Correlation of ASM and ASM/BMI with BMI Z‐score and total body fat percentage. (A and B) Spearman correlation analysis between the appendicular skeletal muscle mass (ASM) and BMI‐Z score (A) and body fat percentage (B) in 1447 children and adolescents (A: *ρ* = −0.019, *p* = 0.474; B: *ρ* = −0.266, *p* < 0.001). (C and D) Spearman correlation analysis between the ASM/BMI and BMI‐Z score (C) and body fat percentage (D) in 1447 children and adolescents (C: *ρ* = −0.363, *p* < 0.474; D: *ρ* = −0.467, *p* < 0.001). BMI, body mass index.
**Figure S2:** Four weeks of high‐fat diet feeding induces impairments in muscle mass and function in juvenile mice. (A–C) Food intake (A), energy intake (B) and protein intake (C) in 3‐week and 7‐week‐old mice over a 24‐h period when consuming 60% HFD or NCD were measured (*n* = 10 mice/group). (D–G) Body composition of juvenile mice at 7 weeks of age: body weight (D), body fat percentage (E), fat‐free mass (F) and total body lean mass (G) (*n* = 10 mice/group). (H) Quantification of average muscle fibre cross‐sectional area (Juvenile‐NCD: *n* = 8, Juvenile‐HFD: *n* = 7). (I and J) Muscle strength assessed by grip strength test in juvenile mice (*n* = 10 mice/group): grip strength normalized to body weight (I) and grip strength normalized to lean mass (J). (K) Motor performance assessed by the rotarod test with maximum speed achieved in juvenile mice (*n* = 10 mice/group). (L and M) Locomotor activity assessed by the open field test in juvenile mice (*n* = 8 mice/group): total distance travelled (L) and average speed (M). Values are presented as mean ± SEM *p < 0.05, **p < 0.01, ***p < 0.001.
**Figure S3:** Four weeks of high‐fat diet feeding induces an obese phenotype but does not affect muscle mass or function in 8‐week‐old adult mice. (A–F) Body composition of adult mice at 12 weeks of age: body weight (A), body fat mass (B), body fat percentage (C), fat‐free mass (D), total body lean mass (E) (*n* = 10 mice/group) and the change in lean mass from 8 to 12 weeks of age (F, *n* = 10 mice/group). (G) Representative H&E‐stained sections of TA muscles from adult mice (scale bar = 100 μm). (H and I) Quantification of muscle fibre diameter (H, *n* = 8 mice/group) and average muscle fibre cross‐sectional area (I, Adult‐NCD: *n* = 8, Adult‐HFD: *n* = 7). (J–L) Muscle strength assessed by grip strength test in adult mice (*n* = 10 mice/group): absolute grip strength (J), grip strength normalized to body weight (K) and grip strength normalized to lean weight (L). (M and N) Motor performance assessed by the rotarod test in adult mice (*n* = 10 mice/group): latency to fall (M) and maximum speed achieved (N). (O and P) Locomotor activity assessed by the open field test in adult mice (*n* = 5 mice/group): total distance travelled (O) and average speed (P). Values are presented as mean ± SEM. *p < 0.05, **p < 0.01, ***p < 0.001.
**Figure S4:** RNA‐seq analysis of TA muscles from adult mice. (A) Heatmap of DEGs in adult HFD‐fed mice compared to adult NCD‐fed mice. (B) Volcano plot depicting 333 upregulated (red) and 521 downregulated (blue) genes in the Adult‐HFD group versus the Adult‐NCD group. (C) GO enrichment analysis of biological processes for the downregulated genes in the Adult‐HFD group compared to the Adult‐NCD group. (D) GO enrichment analysis of biological processes for the 485 adult‐specific downregulated DEGs. Sample size: *n* = 4 mice per group.
**Figure S5:** Age‐dependent transcriptional changes in NCD‐fed mice. (A) Heatmap showing DEGs between Adult‐NCD and Juvenile‐NCD groups. (B) Volcano plot illustrating 560 upregulated (red) and 1086 downregulated (blue) genes in the Adult‐NCD group compared to the Juvenile‐NCD group. (C) GO enrichment analysis of biological processes for the upregulated genes in the Adult‐NCD group compared to the Juvenile‐NCD group. (D) GO enrichment analysis of biological processes for the downregulated genes in the Adult‐NCD group compared to the Juvenile‐NCD group. Sample size: *n* = 4 mice per group.
**Figure S6:** Juvenile HFD‐fed mice displayed increased lipid accumulation in skeletal muscle. (A and B) Representative Oil Red O staining of TA muscle sections (scale bars = 100 μm; *n* = 8 mice/group) and quantification of the stained lipid area. Values are mean ± SEM. *p < 0.05, **p < 0.01, ***p < 0.001.
**Figure S7:** HFD feeding does not alter myogenic or most adipogenic factors in adult mice. (A–D) Gene expression levels in the TA muscle of adult mice (*n* = 8 mice/group unless noted otherwise): (A) mRNA levels of MRFs (for *Myod*, Adult‐HFD: *n* = 7); (B) mRNA levels of MHCs (for *Myh7*, Adult‐HFD: *n* = 7); (C) mRNA levels of potential myogenic regulators; (D) mRNA levels of adipogenic factors (for *Adipsin*, Adult‐NCD: *n* = 6, Adult‐HFD: *n* = 7; for *FABP4*, Adult‐HFD: *n* = 7). Values are mean ± SEM. *p < 0.05, **p < 0.01, ***p < 0.001.
**Figure S8:** Dietary intervention does not reverse HFD‐induced muscle impairments. (A‐D) Body composition and functional assessments at 13 weeks: body fat mass (A), body fat percentage (B), fat‐free mass (C) and total body lean mass (D). (E) Quantification of average muscle fibre cross‐sectional area. (F and G) Muscle strength assessed by grip strength test: grip strength normalized to body weight (F) and grip strength normalized to lean mass (G). (H) Motor performance assessed by the rotarod test with maximum speed achieved. (I and J) Locomotor activity assessed by the open field test: total distance travelled (I) and average speed (J). *n* = 8 mice/group. Values are mean ± SEM. *p < 0.05, **p < 0.01, ***p < 0.001.
**Figure S9:** Vitamin C supplementation alleviates HFD‐induced muscular impairments. (A–D) Body composition and functional assessments at 13 weeks (*n* = 10/group): body fat mass (A), body fat percentage (B), fat‐free mass (C) and total body lean mass (D). (E) Quantification of average muscle fibre cross‐sectional area (*n* = 8/group). (F and G) Muscle strength assessed by grip strength test (*n* = 8/group): grip strength normalized to body weight (F) and grip strength normalized to lean mass (G). (H) Motor performance assessed by the rotarod test with maximum speed achieved (*n* = 8/group). (I and J) Locomotor activity assessed by the open field test (*n* = 10/group): total distance travelled (I) and average speed (J). Data are mean ± SEM. *p < 0.05, **p < 0.01, ***p < 0.001.

**Data S1:** Supporting information.

## Data Availability

The RNA‐Seq data for fresh TA muscle tissues from various groups of C57BL/6J WT mice are available in the GEO database under accession code GSE280926. The data can be accessed at https://www.ncbi.nlm.nih.gov/geo/query/acc.cgi?acc=GSE280926 using the token ‘svyvsgcehfoztaf’. All other relevant data can be found in the Supporting Information.
