# Supplementary material for: Sarcopenic Obesity in Children: An Emerging Complication Evidenced by Clinical Data and a Juvenile Mouse Model
Source: J Cachexia Sarcopenia Muscle. 2026 Apr 17;17(2):e70278. doi: 10.1002/jcsm.70278 (PMC13088153; doi:10.1002/jcsm.70278)
Supplement: Supplementary file 1 — Table S1: RT‐qPCR primer list. Table S2: Characteristic of the participants for muscle mass assessment. Table S3: Characteristic of the participants for muscle function assessment. Figure S1: Correlation of ASM and ASM/BMI with BMI Z‐score and total body fat percentage. (A and B) Spearman correlation analysis between the appendicular skeletal muscle mass (ASM) and BMI‐Z score (A) and body fat percentage (B) in 1447 children and adolescents (A: ρ = −0.019, p = 0.474; B: ρ = −0.266, p < 0.001). (C and D) Spearman correlation analysis between the ASM/BMI and BMI‐Z score (C) and body fat percentage (D) in 1447 children and adolescents (C: ρ = −0.363, p < 0.474; D: ρ = −0.467, p < 0.001). BMI, body mass index. Figure S2: Four weeks of high‐fat diet feeding induces impairments in muscle mass and function in juvenile mice. (A–C) Food intake (A), energy intake (B) and protein intake (C) in 3‐week and 7‐week‐old mice over a 24‐h period when consuming 60% HFD or NCD were measured (n = 10 mice/group). (D–G) Body composition of juvenile mice at 7 weeks of age: body weight (D), body fat percentage (E), fat‐free mass (F) and total body lean mass (G) (n = 10 mice/group). (H) Quantification of average muscle fibre cross‐sectional area (Juvenile‐NCD: n = 8, Juvenile‐HFD: n = 7). (I and J) Muscle strength assessed by grip strength test in juvenile mice (n = 10 mice/group): grip strength normalized to body weight (I) and grip strength normalized to lean mass (J). (K) Motor performance assessed by the rotarod test with maximum speed achieved in juvenile mice (n = 10 mice/group). (L and M) Locomotor activity assessed by the open field test in juvenile mice (n = 8 mice/group): total distance travelled (L) and average speed (M). Values are presented as mean ± SEM *p < 0.05, **p < 0.01, ***p < 0.001. Figure S3: Four weeks of high‐fat diet feeding induces an obese phenotype but does not affect muscle mass or function in 8‐week‐old adult mice. (A–F) Body composition of adult mice at 12 weeks [file JCSM-17-e70278-s002.docx]

**Table S1** RT-qPCR primers list

| Gene Name | Forward Primer | Reverse Primer |
| --- | --- | --- |
| *Gapdh* | ACAACTTTGGCATTGTGGAA | GATGCAGGGATGATGTTCTG |
| *Pax7* | AATCAGCTTGGTGGGGTCTT | ATCGGCACAGAATCTTGGAG |
| *Myod* | GACAGGGAGGAGGGGTAGAG | TGCTGTCTCAAAGGAGCAGA |
| *Myog* | GGTGGAGGATATGTCTGTTG | GTGTTAGCCTTATGTGAATGG |
| *Myf5* | TGACGGCATGCCTGAATGTA | ATCTGCAGCACATGCATTTGATA |
| *Myf6* | TATCACGAGGCCCCTGGAAT | GACTGCCCAAGGTGGAGATT |
| *Myh1* | CTCTTCCCGCTTTGGTAAGTT | CAGGAGCATTTCGATTAGATCCG |
| *Myh2* | GGCTTCAGGATTTGGTGGATAA | GGATCTTGCGGAACTTGGATAG |
| *Myh4* | GATTGACGTGGAGAGGTCTAAC | CCTGAGTTTCCTCGTACTTCTG |
| *Myh7* | GCTGCAGCAGTTCTTCAACC | GGAACATGCACTCCTCCTCA |
| *Btg2* | GGTTGGAGAAAATTGGGAAAC | GCTTCTAAGAAGCCCTCATC |
| *Fos* | TGTTCCTGGCAATAGCGTGT | TCAGACCACCTCGACAATGC |
| *Skil* | GCTGGTTGCTCTCGTAAGGT | CCACCCATAGCAACGTCAGT |
| *Nr4a1* | TGGCTTTGGTGATTGGATTGA | CAATGCGATTCTGCAGCTCT |
| *Adipoq* | TGTTCCTCTTAATCCTGCCCA | CCAACCTGCACAAGTTCCCTT |
| *Adipsin* | CTACATGGCTTCCGTGCAAGT | AGTCGTCATCCGTCACTCCAT |
| *Leptin* | GAGACCCCTGTGTCGGTTC | AGCCCAGGAATGAAGTCCAAG |
| *Fabp4* | GATGAAATCACCGCAGACGAC | ATTCCACCACCAGCTTGTCAC |
| *Ppar-γ* | TCGCTGATGCACTGCCTATG | GAGAGGTCCACAGAGCTGATT |

**Table S2** Characteristics of the participants for muscle mass assessment

| **Variable** | **Overall (N = 1,447)** |
| --- | --- |
| Age (years) | 11.10 (9.39, 12.50) |
| Sex |  |
| Boy | 993.0 (68.6%) |
| Girls | 454.0 (31.4%) |
| Tanner |  |
| 1 | 449.0 (31.0%) |
| 2 | 345.0 (23.8%) |
| 3 | 290.0 (20.0%) |
| 4 | 226.0 (15.6%) |
| 5 | 137.0 (9.5%) |
| Height (cm) | 153.00 (142.40, 161.30) |
| Weight (kg) | 65.00 (52.00, 78.50) |
| BMI (kg/m^2^) | 27.97 (25.54, 31.02) |
| BMI-Z score | 2.93 (2.51, 3.49) |
| Waist circumference (cm) | 89.40 (82.00, 98.00) |
| WHtR | 0.59 (0.56, 0.63) |
| Fat mass (kg) | 6.431 (5.218, 8.230) |
| Body fat percent (%) | 45.50 (41.74, 49.56) |
| Android/Gynoid fat ratio | 1.12 (1.05, 1.18) |
| Fat-free mass (kg) | 14.617 (11.583, 18.218) |
| ASM (kg) | 14.53 (11.55, 18.32) |
| ASMR (%) | 22.68 (20.81, 24.62) |

Data are summarized as medians with interquartile ranges (first, third) for continuous variables and as counts with percentages for categorical ones. BMI, body mass index; WHtR, waist-to-height ratio; ASM, appendicular skeletal muscle mass weight; ASMR, appendicular skeletal muscle mass weight ratio.

**Table S3** Characteristics of the participants for muscle function assessment

| **Variable** | **Overall**  **(N = 349)** | **Normal BMI**  **(N = 93)** | **Obesity**  **(N = 256)** | **p-value** |
| --- | --- | --- | --- | --- |
| Age (years) | 11.13 (9.73, 12.80) | 10.47 (9.00, 11.81) | 11.44 (9.96, 13.02) | **<0.001** |
| Sex |  |  |  | **<0.001** |
| Boy | 233.0 (66.8%) | 42.0 (45.2%) | 191.0 (74.6%) |  |
| Girls | 116.0 (33.2%) | 51.0 (54.8%) | 65.0 (25.4%) |  |
| Height (cm) | 151.50 (141.00, 162.00) | 140.00 (134.00, 147.00) | 155.00 (144.75, 164.00) | **<0.001** |
| Weight (kg) | 57.40 (42.00, 74.00) | 32.00 (27.60, 39.20) | 66.80 (53.05, 80.15) | **<0.001** |
| BMI (kg/m^2^) | 25.70 (20.10, 29.10) | 16.60 (15.10, 18.00) | 27.20 (25.00, 30.50) | **<0.001** |
| BMI-Z score | 2.35 (1.00, 2.88) | -0.11 (-0.72, 0.59) | 2.64 (2.24, 3.06) | **<0.001** |
| Waist circumference (cm) | 83.50 (67.90, 94.00) | 59.00 (54.80, 65.20) | 89.10 (81.00, 96.50) | **<0.001** |
| WHtR | 0.55 (0.47, 0.60) | 0.42 (0.40, 0.44) | 0.58 (0.54, 0.61) | **<0.001** |
| Fat mass (kg) | 22.30 (10.90, 29.70) | 5.40 (2.70, 8.30) | 25.95 (20.55, 32.95) | **<0.001** |
| Body fat percent (%) | 37.50 (27.00, 41.90) | 16.60 (9.30, 21.80) | 39.85 (36.60, 43.30) | **<0.001** |
| Fat-free mass (kg) | 35.30 (28.40, 45.10) | 26.60 (24.10, 29.50) | 38.90 (32.25, 47.60) | **<0.001** |
| Muscle mass (kg) | 32.80 (26.30, 42.00) | 24.70 (22.20, 27.30) | 36.15 (29.95, 44.30) | **<0.001** |
| Muscle percent (%) | 58.20 (54.00, 67.70) | 77.10 (72.50, 84.10) | 55.90 (52.70, 58.85) | **<0.001** |
| Handgrip strength (kg) | 14.50 (9.90, 21.40) | 10.10 (7.90, 14.10) | 16.10 (11.45, 22.80) | **<0.001** |
| Adjusted grip strength per muscle mass (kg/kg)* | / | 0.782 (0.719, 0.846) | 0.772 (0.739, 0.804) | 0.803 |

Data are summarized as medians with interquartile ranges (first, third) for continuous variables and as counts with percentages for categorical ones. The Mann-Whitney U test and Fisher’s exact test were employed to analyze continuous and categorical variables, respectively. Statistical significance (P < 0.05) is indicated by bold type. BMI, body mass index; WHtR, waist-to-height ratio.

* Adjusted grip strength per unit of muscle mass was obtained through analysis of covariance (ANCOVA), controlling for age and sex, and presented as adjusted means with 95% confidence intervals for the two groups.


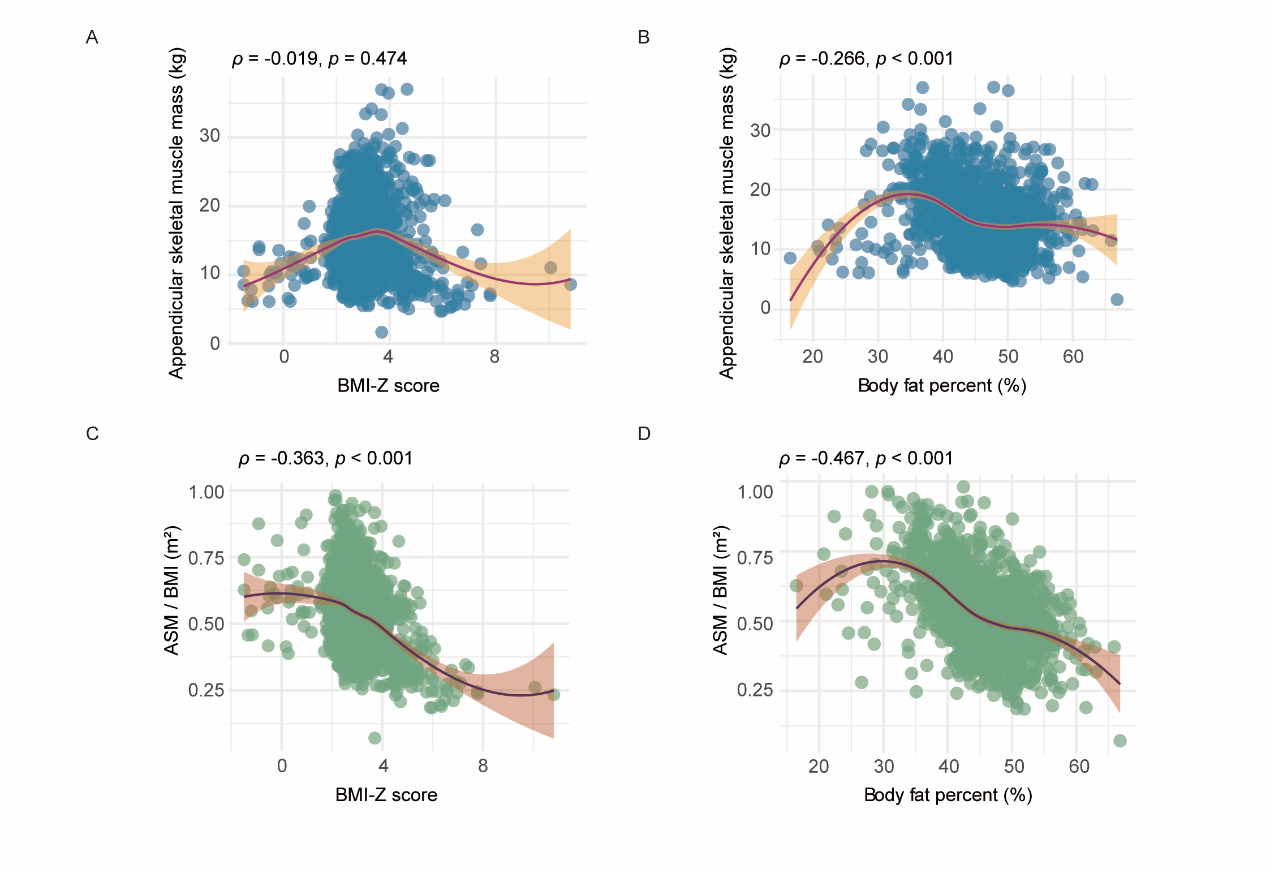


**Figure S1** Correlation of ASM and ASM/BMI with BMI Z-score and total body fat percentage. (A and B) Spearman correlation analysis between the appendicular skeletal muscle mass (ASM) and BMI-Z score (A), and body fat percentage (B) in 1,447 children and adolescents (A: ρ = -0.019, p = 0.474; B: ρ = -0.266, p < 0.001). (C and D) Spearman correlation analysis between the ASM/BMI and BMI-Z score (C), and body fat percentage (D) in 1,447 children and adolescents (C: ρ = -0.363, p < 0.474; D: ρ = -0.467, p < 0.001). BMI, body mass index.


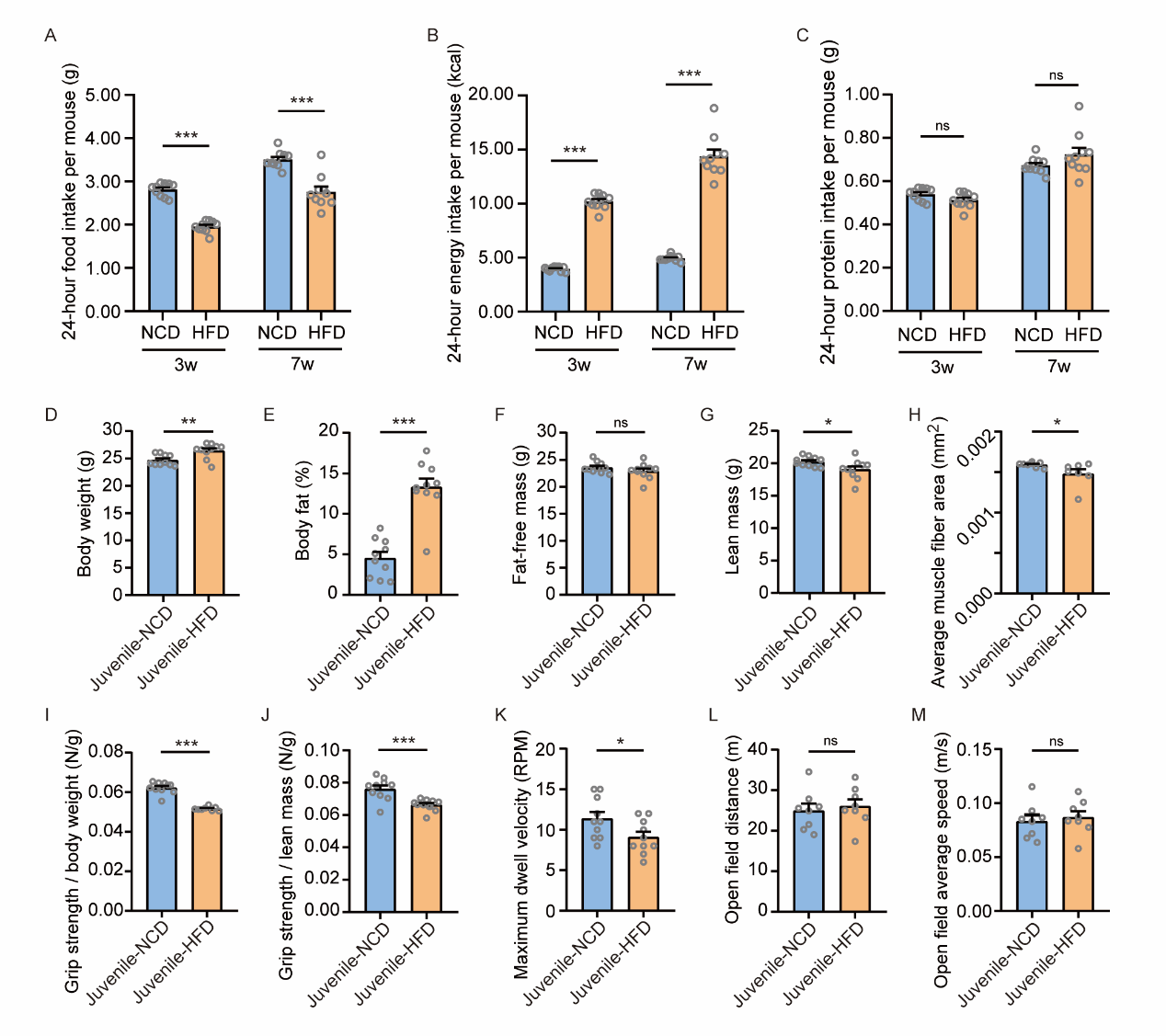


Figure S2 Four weeks of high-fat diet feeding induces impairments in muscle mass and function in juvenile mice. (A-C) Food intake (A), energy intake (B) and protein intake (C) in 3-week and 7-week-old mice over a 24-hour period when consuming 60% HFD or NCD were measured (n = 10 mice/group). (D-G) Body composition of juvenile mice at 7 weeks of age: body weight (D), body fat percentage (E), fat-free mass (F), and total body lean mass (G) (n = 10 mice/group). (H) Quantification of average muscle fiber cross-sectional area (Juvenile-NCD: n=8, Juvenile-HFD: n=7). (I and J) Muscle strength assessed by grip strength test in juvenile mice (n = 10 mice/group): grip strength normalized to body weight (I), and grip strength normalized to lean mass (J). (K) Motor performance assessed by the rotarod test with maximum speed achieved in juvenile mice (n = 10 mice/group). (L and M) Locomotor activity assessed by the open field test in juvenile mice (n = 8 mice/group): total distance traveled (L) and average speed (M). Values are presented as mean ± SEM. *p < 0.05, **p < 0.01, ***p < 0.001.


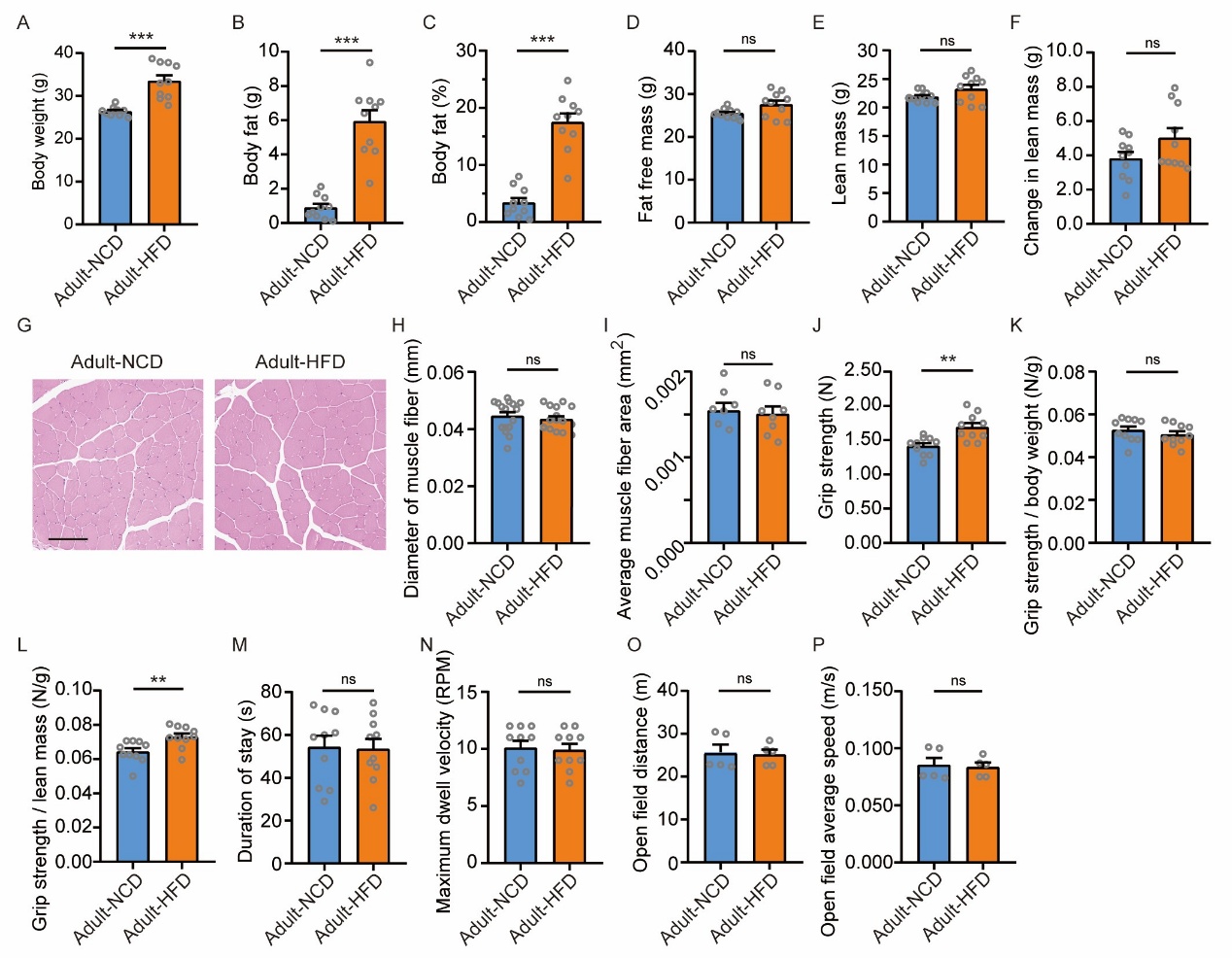


Figure S3 Four weeks of high-fat diet feeding induces an obese phenotype but does not affect muscle mass or function in 8-week-old adult mice. (A-F) Body composition of adult mice at 12 weeks of age: body weight (A), body fat mass (B), body fat percentage (C), fat-free mass (D), total body lean mass (E) (n = 10 mice/group), and the change in lean mass from 8 to 12 weeks of age (F, n = 10 mice/group). (G) Representative H&E-stained sections of TA muscles from adult mice (scale bar = 100 µm). (H and I) Quantification of muscle fiber diameter (H, n=8 mice/group) and average muscle fiber cross-sectional area (I, Adult-NCD: n=8, Adult-HFD: n=7). (J-L) Muscle strength assessed by grip strength test in adult mice (n = 10 mice/group): absolute grip strength (J), grip strength normalized to body weight (K), and grip strength normalized to lean weight (L). (M and N) Motor performance assessed by the rotarod test in adult mice (n = 10 mice/group): latency to fall (M) and maximum speed achieved (N). (O and P) Locomotor activity assessed by the open field test in adult mice (n = 5 mice/group): total distance traveled (O) and average speed (P). Values are presented as mean ± SEM. *p < 0.05, **p < 0.01, ***p < 0.001.


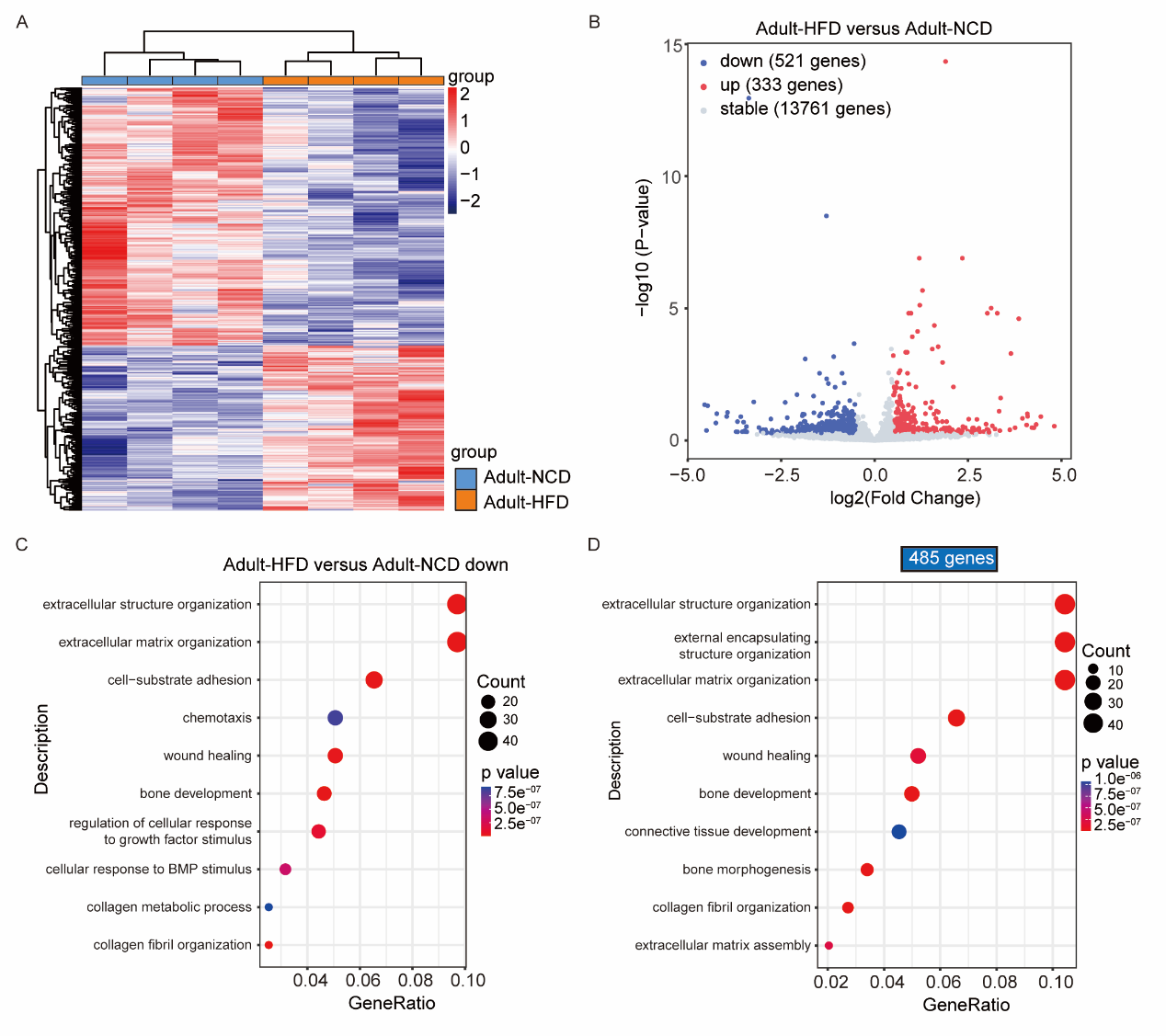


**Figure S4** RNA-seq analysis of TA muscles from adult mice. (A) Heatmap of DEGs in adult HFD-fed mice compared to adult NCD-fed mice. (B) Volcano plot depicting 333 upregulated (red) and 521 downregulated (blue) genes in the Adult-HFD group versus the Adult-NCD group. (C) GO enrichment analysis of biological processes for the downregulated genes in the Adult-HFD group compared to the Adult-NCD group. (D) GO enrichment analysis of biological processes for the 485 adult-specific downregulated DEGs. Sample size: n = 4 mice per group.


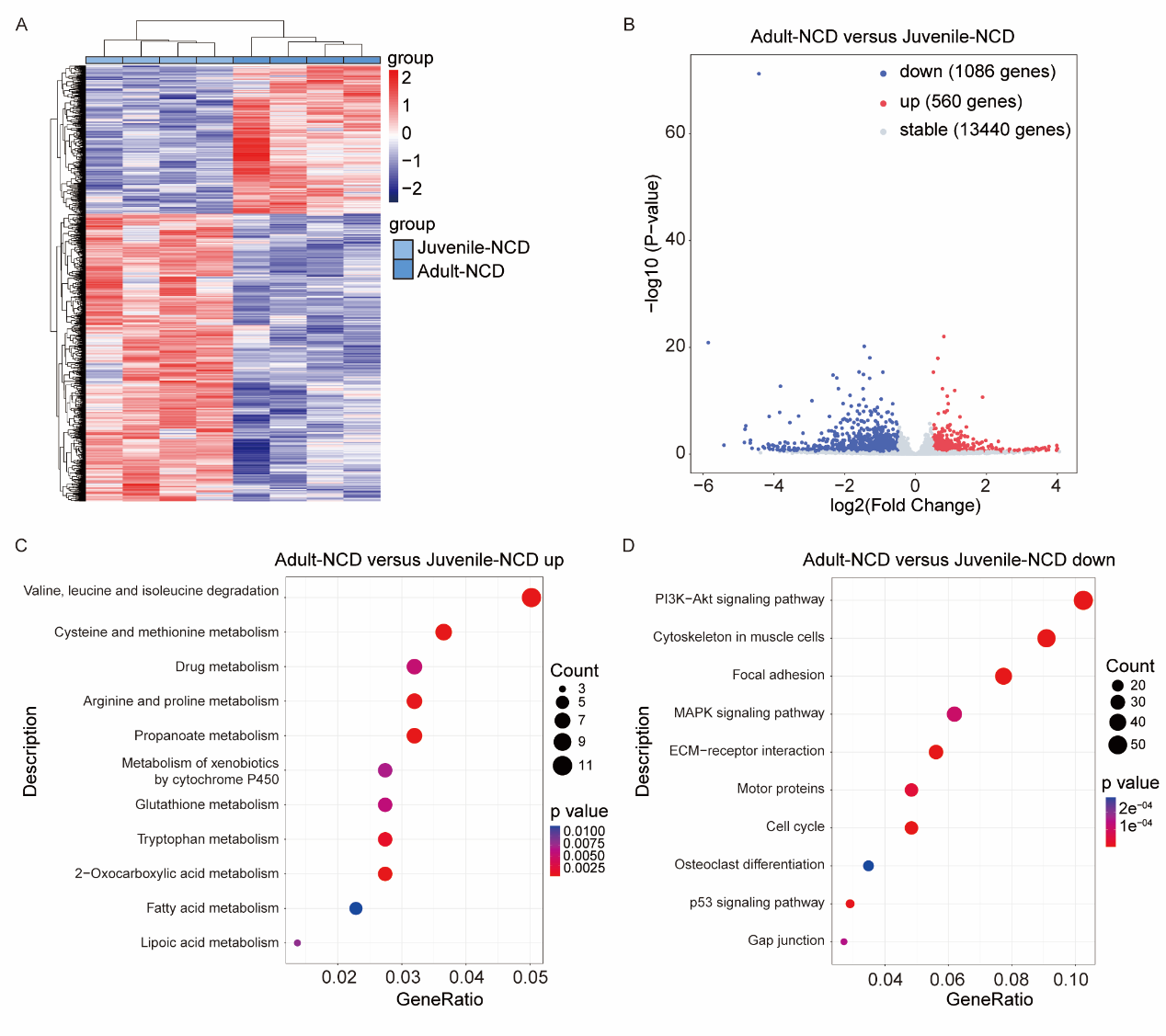


**Figure S5** Age-dependent transcriptional changes in NCD-fed mice. (A) Heatmap showing DEGs between Adult-NCD and Juvenile-NCD groups. (B) Volcano plot illustrating 560 upregulated (red) and 1086 downregulated (blue) genes in the Adult-NCD group compared to the Juvenile-NCD group. (C) GO enrichment analysis of biological processes for the upregulated genes in the Adult-NCD group compared to the Juvenile-NCD group. (D) GO enrichment analysis of biological processes for the downregulated genes in the Adult-NCD group compared to the Juvenile-NCD group. Sample size: n = 4 mice per group.


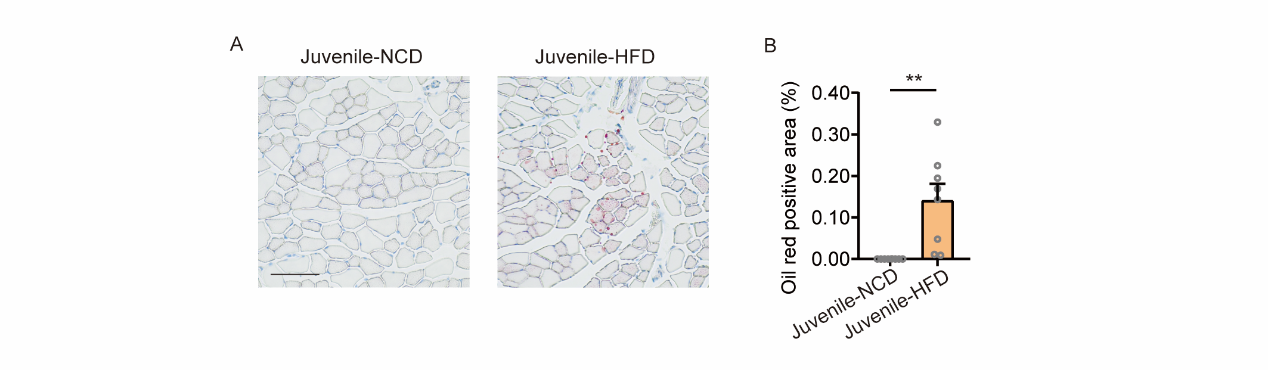


Figure S6 Juvenile HFD-fed mice displayed increased lipid accumulation in skeletal muscle. (A and B) Representative Oil Red O staining of TA muscle sections (scale bars = 100 μm; n = 8 mice/group) and quantification of the stained lipid area. Values are mean ± SEM. *p < 0.05, **p < 0.01, ***p < 0.001.


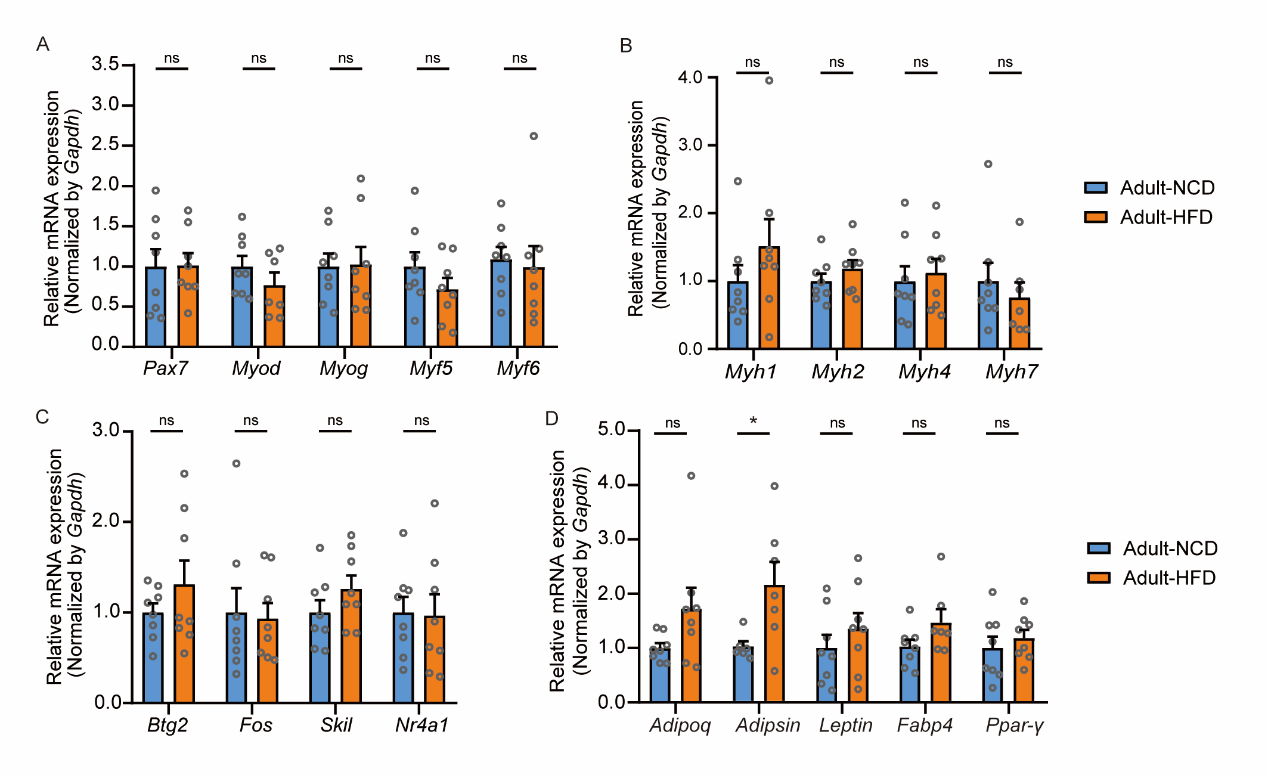


**Figure S7** HFD feeding does not alter myogenic or most adipogenic factors in adult mice. (A–D) Gene expression levels in the TA muscle of adult mice (n = 8 mice/group unless noted otherwise): (A) mRNA levels of MRFs (for *Myod*, Adult-HFD: n = 7); (B) mRNA levels of MHCs (for *Myh7*, Adult-HFD: n = 7); (C) mRNA levels of potential myogenic regulators; (D) mRNA levels of adipogenic factors (for *Adipsin*, Adult-NCD: n = 6, Adult-HFD: n = 7; for *FABP4*, Adult-HFD: n=7). Values are mean ± SEM. *p < 0.05, **p < 0.01, ***p < 0.001.


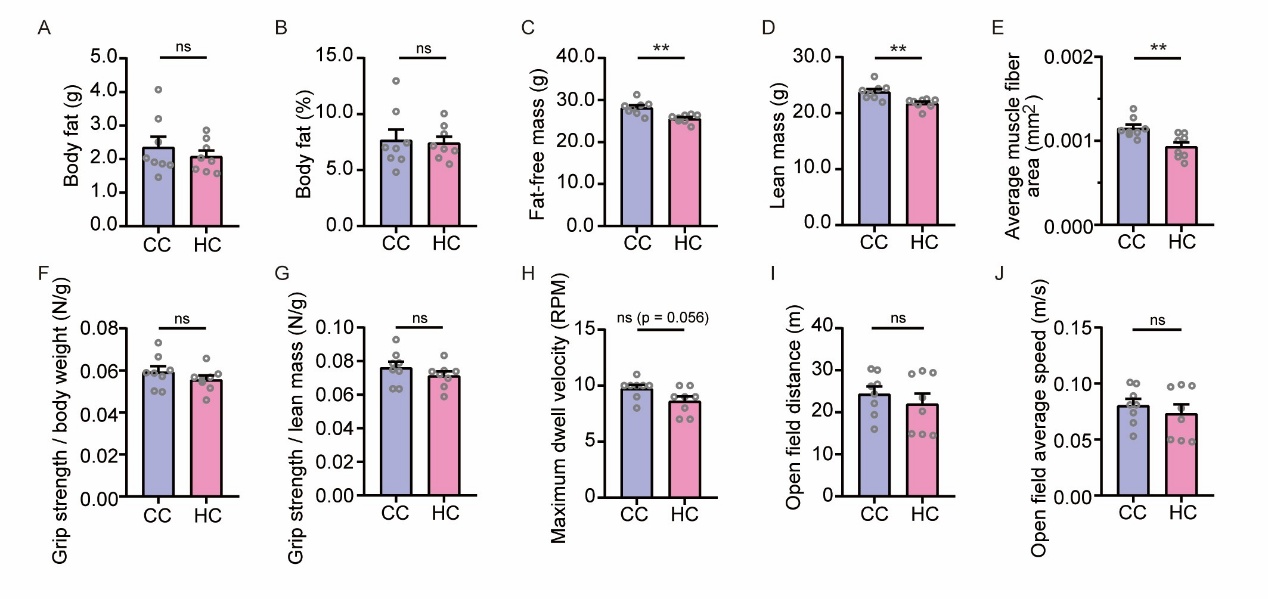


Figure S8 Dietary intervention does not reverse HFD-induced muscle impairments. (A-D) Body composition and functional assessments at 13 weeks: body fat mass (A), body fat percentage (B), fat-free mass (C), and total body lean mass (D). (E) Quantification of average muscle fiber cross-sectional area. (F and G) Muscle strength assessed by grip strength test: grip strength normalized to body weight (F), and grip strength normalized to lean mass (G). (H) Motor performance assessed by the rotarod test with maximum speed achieved. (I and J) Locomotor activity assessed by the open field test: total distance traveled (I) and average speed (J). n = 8 mice/group. Values are mean ± SEM. *p < 0.05, **p < 0.01, ***p < 0.001.


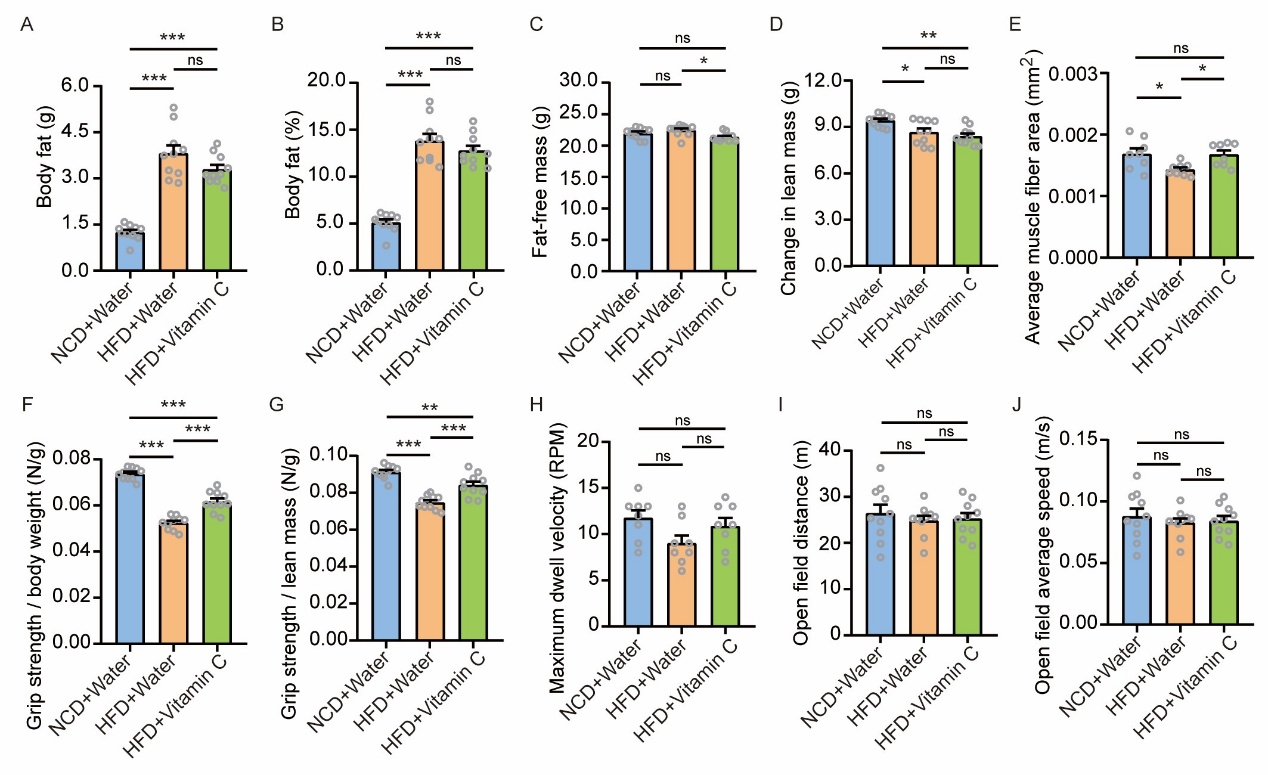


Figure S9 Vitamin C supplementation alleviates HFD-induced muscular impairments. (A-D) Body composition and functional assessments at 13 weeks (n=10/group): body fat mass (A), body fat percentage (B), fat-free mass (C), and total body lean mass (D). (E) Quantification of average muscle fiber cross-sectional area (n = 8/group). (F and G) Muscle strength assessed by grip strength test (n = 8/group): grip strength normalized to body weight (F), and grip strength normalized to lean mass (G). (H) Motor performance assessed by the rotarod test with maximum speed achieved (n = 8/group). (I and J) Locomotor activity assessed by the open field test (n = 10/group): total distance traveled (I) and average speed (J). Data are mean ± SEM. *p < 0.05, **p < 0.01, ***p < 0.001.

**Supplementary references**

[S1] De Onis M; Onyango A W; Borghi E, et al.Development of a WHO growth reference for school-aged children and adolescents.*Bull World Health Organ*,**2007**, 85 (9): 660-7.<https://doi.org/10.2471/blt.07.043497>.

[S2] Marshall W A; Tanner J M.Variations in the pattern of pubertal changes in boys.*Arch Dis Child*,**1970**, 45 (239): 13-23.<https://doi.org/10.1136/adc.45.239.13>.

[S3] Cruz-Jentoft A J; Baeyens J P; Bauer J M, et al.Sarcopenia: European consensus on definition and diagnosis: Report of the European Working Group on Sarcopenia in Older People.*Age Ageing*,**2010**, 39 (4): 412-23.<https://doi.org/10.1093/ageing/afq034>.

[S4] China N H a F P C O T P S R O. Screening for overweight and obesity among school-age children and adolescents: WS/T 586-2018[S].National Health and Family Planning Commission of the People's Republic of China,2018.

[S5] Wang C; Yue F; Kuang S.Muscle Histology Characterization Using H&E Staining and Muscle Fiber Type Classification Using Immunofluorescence Staining.*Bio Protoc*,**2017**, 7 (10).<https://doi.org/10.21769/BioProtoc.2279>.

[S6] Mehlem A; Hagberg C E; Muhl L, et al.Imaging of neutral lipids by oil red O for analyzing the metabolic status in health and disease.*Nat Protoc*,**2013**, 8 (6): 1149-54.<https://doi.org/10.1038/nprot.2013.055>.

[S7] Li C; Yan Y; Cheng J, et al.Toll-Like Receptor 4 Deficiency Causes Reduced Exploratory Behavior in Mice Under Approach-Avoidance Conflict.*Neurosci Bull*,**2016**, 32 (2): 127-36.<https://doi.org/10.1007/s12264-016-0015-z>.

[S8] Dobin A; Davis C A; Schlesinger F, et al.STAR: ultrafast universal RNA-seq aligner.*Bioinformatics*,**2013**, 29 (1): 15-21.<https://doi.org/10.1093/bioinformatics/bts635>.

[S9] Liao Y; Smyth G K; Shi W.featureCounts: an efficient general purpose program for assigning sequence reads to genomic features.*Bioinformatics*,**2014**, 30 (7): 923-30.<https://doi.org/10.1093/bioinformatics/btt656>.

[S10] Frankish A; Diekhans M; Ferreira A M, et al.GENCODE reference annotation for the human and mouse genomes.*Nucleic Acids Res*,**2019**, 47 (D1): D766-d773.<https://doi.org/10.1093/nar/gky955>.

[S11] Bardou P; Mariette J; Escudié F, et al.jvenn: an interactive Venn diagram viewer.*BMC Bioinformatics*,**2014**, 15 (1): 293.<https://doi.org/10.1186/1471-2105-15-293>.

[S12] Mishra M; Kane A E; Young A P, et al.Age, sex, and frailty modify the expression of common reference genes in skeletal muscle from ageing mice.Mech Ageing Dev,2023, 210: 111762.https://doi.org/10.1016/j.mad.2022.111762.

[S13] Fan X; Yao H; Liu X, et al.High-Fat Diet Alters the Expression of Reference Genes in Male Mice.Front Nutr,2020, 7: 589771.https://doi.org/10.3389/fnut.2020.589771.

[S14] Billich N; Adams J; Carroll K, et al.The Relationship between Obesity and Clinical Outcomes in Young People with Duchenne Muscular Dystrophy.Nutrients,2022, 14 (16).https://doi.org/10.3390/nu14163304.

[S15] Goldman V; Ryabets-Lienhard A; Howard L, et al.Obesity Management in Youth with Duchenne Muscular Dystrophy: A Review of Metformin and Alternative Pharmacotherapies.Child Obes,2025, 21 (2): 103-112.https://doi.org/10.1089/chi.2024.0297.

[S16] Deheuninck J; Luo K.Ski and SnoN, potent negative regulators of TGF-beta signaling.*Cell Res*,**2009**, 19 (1): 47-57.<https://doi.org/10.1038/cr.2008.324>.

[S17] Liu D; Black B L; Derynck R.TGF-beta inhibits muscle differentiation through functional repression of myogenic transcription factors by Smad3.*Genes Dev*,**2001**, 15 (22): 2950-66.<https://doi.org/10.1101/gad.925901>.

[S18] Wrighton K H; Liang M; Bryan B, et al.Transforming growth factor-beta-independent regulation of myogenesis by SnoN sumoylation.*J Biol Chem*,**2007**, 282 (9): 6517-24.<https://doi.org/10.1074/jbc.M610206200>.

[S19] Pette D; Staron R S.Myosin isoforms, muscle fiber types, and transitions.*Microsc Res Tech*,**2000**, 50 (6): 500-9.<https://doi.org/10.1002/1097-0029(20000915)50:6><500::Aid-jemt7>3.0.Co;2-7.

[S20] Emerald B S; Al Jailani M A; Ibrahim M F, et al.Cellular and Molecular Variations in Male and Female Murine Skeletal Muscle after Long-Term Feeding with a High-Fat Diet.Int J Mol Sci,2022, 23 (17).https://doi.org/10.3390/ijms23179547.

[S21] Ullah R; Shen Y; Zhou Y D, et al.Perinatal metabolic inflammation in the hypothalamus impairs the development of homeostatic feeding circuitry.*Metabolism*,**2023**, 147: 155677.<https://doi.org/10.1016/j.metabol.2023.155677>.

[S22] Iglesias P.Muscle in Endocrinology: From Skeletal Muscle Hormone Regulation to Myokine Secretion and Its Implications in Endocrine-Metabolic Diseases.J Clin Med,2025, 14 (13).https://doi.org/10.3390/jcm14134490.

[S23] Ghanim H; Dhindsa S; Batra M, et al.Effect of Testosterone on FGF2, MRF4, and Myostatin in Hypogonadotropic Hypogonadism: Relevance to Muscle Growth.J Clin Endocrinol Metab,2019, 104 (6): 2094-2102.https://doi.org/10.1210/jc.2018-01832.
